# Power-drill fluoroscopy-controlled technique as an alternative to navigation-assisted pedicle screw placement: A propensity score-matched retrospective cohort study

**DOI:** 10.1016/j.bas.2026.105959

**Published:** 2026-02-02

**Authors:** Felix Corr, Faizan Kareem, Linda Bättig, Benedict Caspari, Karl Kapahnke, Silvio Heinig, Simon Behringer, Erik Schulz, Yesim Yildiz, Nader Hejrati, Oliver Bozinov, Benjamin Martens, Kern Singh, Martin N. Stienen, Stefan Motov

**Affiliations:** a Department of Neurosurgery & Interdisciplinary Spine Center, HOCH Health Ostschweiz, Cantonal Hospital St. Gallen, Rorschacher Strasse 95, 9007, St. Gallen, Switzerland; b Faculty of Medicine, University of Zürich, Rämistrasse 100, 8006, Zürich, Switzerland; c Faculty of Medicine, University of Düsseldorf, Universitätsstrasse 1, 40225, Düsseldorf, Germany; d Faculty of Medicine, University of Greifswald, Domstrasse 11, 17489, Greifswald, Germany; e Department of Orthopedic Surgery and Traumatology & Interdisciplinary Spine Center, HOCH Health Ostschweiz, Cantonal Hospital St. Gallen, St. Gallen, Switzerland; f Department of Orthopaedic Surgery, Rush University Medical Center, 1611 W. Harrison St., Suite #300, Chicago, IL, 60612, USA

**Keywords:** Image-guided surgery, Navigation-assisted surgery, Pedicle screws, Propensity score, Radiation exposure, Spinal fusion

## Abstract

**Introduction:**

Power-drill fluoroscopy-controlled freehand (PFH) pedicle screw placement is a drill-based refinement of the freehand technique that combines fluoroscopic control with tactile feedback. Its performance in degenerative spine surgery remains underexplored. This study compared the accuracy and procedural characteristics of PFH and navigation-assisted (NA) screw placement in thoracolumbar posterior fusion.

**Research question:**

To investigate whether PFH pedicle screw placement achieves comparable accuracy to NA techniques in degenerative thoracolumbar fusion surgery.

**Material and methods:**

Adults undergoing elective thoracolumbar fusion with PFH or NA pedicle screw placement were retrospectively analyzed. Propensity score matching balanced demographic and clinical variables between groups. Screw accuracy was graded by the Gertzbein–Robbins Scale (GRS). Secondary outcomes included radiation exposure and perioperative variables. A generalized linear mixed model accounted for multiple screws per patient.

**Results:**

After matching, 35 patients per group were analyzed (PFH, 224 screws; NA, 154 screws). Satisfactory screw placement was achieved in 97.8% of PFH and 93.5% of NA screws (p = 0.02). Revision rates within 12 months were similar (5.7% vs 17.1%; p = 0.26). Radiation exposure was lower with PFH (8378 ± 5302 vs 23,793 ± 13,162 mGy cm^2^; p < 0.001), despite longer constructs and more frequent cement utilization. Osteoporosis independently reduced accuracy (OR 0.43; 95% CI, 0.20–0.92; p = 0.02).

**Discussion and conclusion:**

In this propensity-matched analysis, PFH demonstrated accuracy comparable to NA (absolute difference 4.3 percentage points) with significantly lower patient radiation exposure. Further investigations are needed to justify the PFH method as a potential alternative in selected degenerative cases where radiation reduction is prioritized or navigation is unavailable.

## Highlights

• Power-drill freehand technique tested against navigation for spinal screw placement.
• Both techniques achieved high accuracy in degenerative thoracolumbar fusion.
• Freehand power-drill placement significantly reduced patient radiation exposure.
• Screw accuracy was comparable between the power-drill and navigation-assisted methods.
• Findings support power-drill freehand as a valid alternative in selected degenerative cases.

## Abbreviations

ASA –: American Society of Anesthesiologists
BMI –: Body Mass Index
CCI –: Charlson Comorbidity Index
CT –: Computed Tomography
DAP –: Dose–Area Product
GRS –: Gertzbein–Robbins Scale
MAR –: Missing at Random
NA –: Navigation-Assisted
PFH –: Power-Drill Fluoroscopy-Controlled Freehand
PSM –: Propensity Score Matching
SD –: Standard Deviation
SMD –: Standardized Mean Difference
TDN –: Therapy–Disability–Neurology

## 1 Introduction

Pedicle screw instrumentation remains the cornerstone of tricolumnar spinal stabilization, providing biomechanical stability while withstanding substantial corrective forces (Roy-Camille et al., 1986). Suboptimal screw placement compromises construct stability, increasing mechanical stress (Yang et al., 2023), material fatigue (Chu et al., 2019), and the chance for neurovascular injuries (Jutte and Castelein, 2002), potentially leading to costly revisions with added complication burdens.

Navigation-assisted (NA) techniques have become increasingly adopted in spine surgery, particularly for complex procedures including deformity correction, tumor resection, and revision cases where anatomical landmarks may be obscured or distorted (Dominy et al., 2022a; Motov et al., 2025). These systems provide real-time three-dimensional visualization to guide screw trajectory planning and aim to reduce occupational radiation exposure to the surgical team (Sippel et al., 2024). However, NA techniques require substantial capital investment, introduce workflow complexity, and remain vulnerable to registration errors from intersegmental motion or intraoperative anatomical shifts (Glossop and Hu, 1997; Guha et al., 2019; Dominy et al., 2022b). Despite these considerations, NA has demonstrated high accuracy rates in published series and represents an important technological advancement in spine surgery (Gelalis et al., 2012; Papalia et al., 2024).

The majority of pedicle screw placements, however, occur in routine degenerative pathology with preserved spinal anatomy, where the incremental benefit of navigation over systematic manual techniques remains unclear. Traditional freehand insertion relies exclusively on anatomical landmark identification and manual pedicle probing, a technique-dependent approach that has demonstrated variable accuracy across surgeons and anatomical levels. Power-drill fluoroscopy-controlled freehand (PFH) represents a technically distinct drill-based method that combines systematic workflow with mechanical advantages not present in conventional manual techniques (Faldini et al., 2021). Unlike manual awl probing, PFH utilizes a thin, flexible power drill bit to navigate the pedicle trajectory, potentially offering improved centering within the pedicle canal, reduced insertion wobble, and enhanced tactile feedback during cortical palpation (Claeson et al., 2022; Skaggs et al., 2020; Yan et al., 2018). These technical characteristics distinguish PFH from traditional freehand approaches and may influence trajectory control, procedural efficiency, and clinical outcomes.

Although PFH has demonstrated high accuracy in deformity and pediatric scoliosis cohorts (Faldini et al., 2024), its performance in common degenerative spinal conditions remains unassessed. To our knowledge, no study has evaluated PFH versus NA screw placement in adult degenerative thoracolumbar surgery. Understanding the relative accuracy, safety profile, and procedural characteristics of these approaches in degenerative cases is clinically relevant, particularly in settings where navigation resources may be limited or where radiation reduction is prioritized. We conducted a propensity score-matched retrospective cohort study to compare the accuracy and clinical outcomes of PFH versus NA pedicle screw placement in patients undergoing surgery for degenerative thoracolumbar pathology. We hypothesized that PFH would demonstrate non-inferior screw placement accuracy compared to NA techniques in this patient population.

## 2 Materials and methods

### 2.1 Study design and setting

This retrospective, single-center cohort study was conducted at the tertiary interdisciplinary spine center, HOCH Health Ostschweiz, Cantonal Hospital St. Gallen, Switzerland. Patients were recruited between January 2022 and February 2024, with follow-up through February 2025. The study followed the Strengthening Reporting of Observational Studies in Epidemiology (STROBE) criteria (Elm et al., 2008) (see Appendix, Table A1). Approval was obtained from the regional ethics committee of Eastern Switzerland (BASEC ID, 2023–01343).

### 2.2 Study population

Adults (≥18 years) who underwent elective thoracic and/or lumbar pedicle screw placement and had postoperative computed tomography (CT) of the relevant spinal region were eligible. The cohort included only patients with degenerative spinal disease (e.g., disc herniation, osteochondrosis, spinal stenosis with instability, degenerative or isthmic spondylolisthesis) and excluded cases of adjacent segment degeneration. Patients were excluded if postoperative CT was unavailable, if fixation involved both PFH and NA techniques, or if an in–out–in screw trajectory was preplanned. Eligible cases were retrospectively identified through the institutional electronic database using procedural and diagnostic codes. Postoperative imaging and clinical records were reviewed to confirm inclusion. CT imaging was obtained either as part of routine follow-up or clinical assessment, as detailed in Supplementary Table S1.

### 2.3 Implant systems and surgical technique

Instrumentation comprised fenestrated, cannulated pedicle screws (MOSS®, Biedermann Technologies, Donaueschingen, Germany; EXPEDIUM VERSE®, DePuy Synthes, Raynham, MA, USA). Pedicle screw augmentation was performed using either PFH or NA according to standardized institutional protocols. All procedures were conducted by board-certified spine surgeons following uniform operative techniques to maintain procedural consistency.

The general PFH technique has been described before (Faldini et al., 2021). In this study, it was combined with fluoroscopic trajectory control as part of our institutional workflow. After standard posterior midline exposure and confirmation of the target vertebral level, the posterior bony anatomy was exposed subperiosteally, including the spinous processes, laminae, facet joints, and pedicle entry regions. The pedicle entry point was identified using established anatomical landmarks (Magerl technique (Dick et al., 1985)). The cortical surface of the pedicle was initially breached using an awl to define the entry point and to prevent skiving of subsequent instrumentation. Pedicle cannulation was then initiated using a 2.0-mm flexible power drill (Colibri®, DePuy Synthes). Drilling was performed in short, controlled bursts without the application of axial pressure, allowing the drill bit to advance autonomously along the path of least resistance within the cancellous bone. The high flexibility of the thin drill bit permits slight elastic bending along the pedicle cortex while promoting natural self-centering within the pedicle channel, thereby reducing lateral wobble and uncontrolled deviation. Pedicle perforation over approximately the first 10–15 mm was achieved using this technique.

Advancement was continued incrementally under tactile control until resistance consistent with the anterior vertebral cortex was perceived. At this point, active drilling was stopped, and mechanical back-and-forth probing without further advancement was performed to assess depth and confirm anterior cortical integrity. This step was intended to provide depth awareness and prevent unintended anterior cortical perforation rather than to deliberately breach or engage the anterior cortex.

Following creation of the pilot tract, a short guidewire (stick) was inserted into the prepared trajectory. Fluoroscopy was then obtained in anteroposterior and lateral projections to verify pedicle trajectory, centering within the pedicle, mediolateral orientation relative to the spinal canal and recess, and depth. Fluoroscopic stick control served as an active decision point in the workflow. If the trajectory was judged satisfactory, the procedure proceeded to definitive instrumentation. If fluoroscopy demonstrated suboptimal alignment, such as medialization toward the spinal canal or recess, lateral deviation, or insufficient centering within the pedicle, the trajectory was corrected prior to screw insertion.

Trajectory correction was performed through the same cortical entry point using a stiffer 2.8-mm drill bit. In contrast to the flexible 2.0-mm drill, the increased stiffness of the 2.8-mm drill allows controlled redirection of the pilot tract by applying targeted pressure in the desired direction. The corrected trajectory was estimated based on fluoroscopic imaging, the orientation of the sticks, and anatomical landmarks. This step reforms the cancellous channel along the corrected vector and reduces the likelihood of re-entering the initial, undesired tract during subsequent screw insertion. Trajectory correction was performed selectively and only when fluoroscopic verification indicated deviation. After correction, we usually advanced with screw placement, but could be reinserted and repeat fluoroscopic control in anteroposterior and lateral views could be obtained to confirm satisfactory alignment and depth, if in doubt. In this workflow, fluoroscopy is intentionally used as an intraoperative trajectory control and correction tool rather than as continuous real-time guidance during drilling. The final screw trajectory is therefore determined by the combination of anatomical landmark identification, tactile feedback during drilling, and fluoroscopic verification with the opportunity for immediate correction before definitive screw placement.

**Fig. 1:**
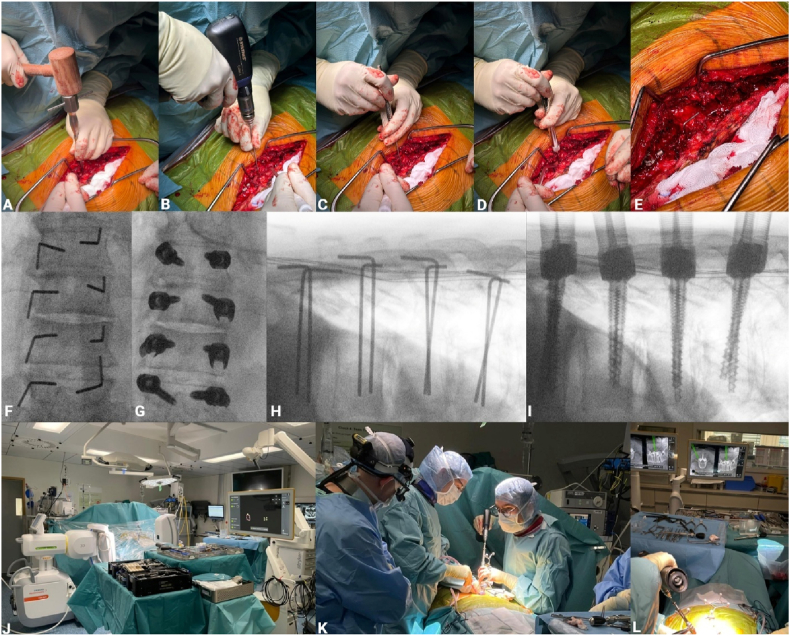
Surgical workflow of PFH and NA pedicle-screw placement. (A) Initial perforation of the pedicle using an awl to mark the entry point. (B) Drilling of the pedicle trajectory using a 2.0 mm flexible power-drill. (C) Tactile probing of the anterior vertebral cortex to assess depth and ensure cortical integrity. (D) Depth measurement using a length probe for trajectory verification. (E) Insertion of a guidewire into the established pedicle trajectory. (F) Anteroposterior fluoroscopic image confirming proper stick alignment in all target pedicles. (G) Anteroposterior view showing final pedicle screw placement. (H) Lateral fluoroscopic image verifying stick positioning along the pedicle axis. (I) Lateral view of final screw placement demonstrating correct alignment and depth. (J) Intraoperative setup for the navigated (NA) technique using 3D imaging and navigation system. (K + J) Real-time navigated pedicle screw placement guided by a tracked drill guide.

Pedicle screw placement in the navigation-assisted (NA) group was performed using an optical neuronavigation system (Curve™, Brainlab AG, Munich, Germany) in combination with intraoperative three-dimensional (3D) fluoroscopic imaging (Cios Spin®, Siemens Healthineers). All procedures were conducted through a standard posterior midline approach with the patient positioned prone on a radiolucent operating table. After subperiosteal dissection, the posterior bony anatomy was exposed in a standardized fashion, including the spinous processes, laminae, facet joints, and the medial aspects of the transverse processes. Care was taken to achieve wide and symmetric exposure to facilitate reliable landmark verification and stable reference array fixation. A rigid navigation reference frame was then attached to the most caudal exposed spinous process within, or immediately adjacent to, the instrumented segment using a clamp, ensuring firm fixation without perceptible micromotion. The caudal anchoring site was selected to minimize interference with surgical instrumentation and to reduce the likelihood of reference frame displacement during multilevel instrumentation. Following sterile draping, an intraoperative 3D scan was acquired using the Cios Spin® system. The acquired dataset was automatically transferred to the navigation workstation and registered to the patient anatomy. Prior to pedicle preparation, navigation accuracy was systematically verified by registering multiple exposed anatomical landmarks (spinous process tips, laminar edges, facet joints) using a navigated pointer. Any discrepancy between the virtual and physical anatomy prompted reassessment of reference array stability and, if necessary, repetition of the 3D scan and registration. All navigated instruments, including the drill guide, were calibrated and registered according to the manufacturer's protocol and re-verified immediately before use. For each pedicle, the optimal entry point and screw trajectory were planned using the navigated pointer in axial, sagittal, and coronal views. A tracked 3.2-mm drill guide was positioned precisely at the planned entry point and aligned with the intended pedicle axis under real-time navigation feedback. Using a Colibri® power drill (DePuy Synthes) equipped with a 3.2-mm drill bit, the transpedicular pilot tract was created to the desired depth under continuous navigational visualization. Immediately after drilling, a K-wire was advanced through the drill guide into the prepared trajectory to preserve alignment and prevent trajectory deviation during subsequent steps. For both techniques, pedicle integrity was assessed using a pedicle probe to exclude cortical breach before screw placement. A guidewire was inserted, followed by a cannulated tap (under-tapping by 1 mm) and the pedicle screw using a cannulated screwdriver. Fluoroscopic a. p./lateral or 3D control of the screws was performed after placement of all implants, depending on the surgeon's preference and available C-arm. The surgical workflow for both techniques is illustrated in Fig. 1.

### 2.4 Quality assessment of screw placement accuracy

**Fig. 2:**
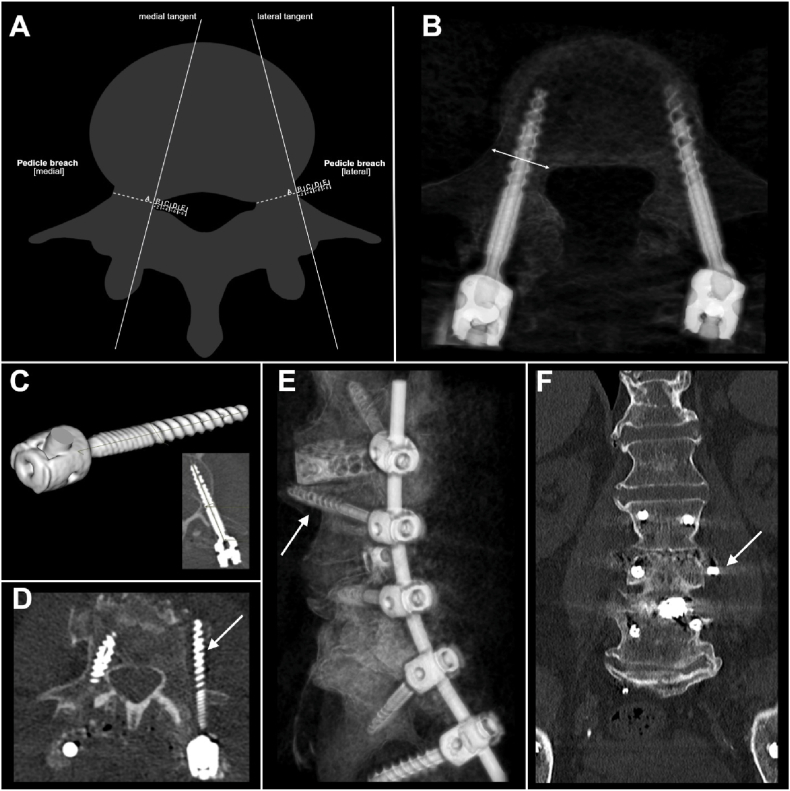
Pedicle screw placement accuracy and breach assessment. (A) Schematic representation of the GRS classification, illustrating medial and lateral pedicle breaches relative to the medial and lateral pedicle walls. (B) Axial CT scan demonstrating the spatial relationship between the pedicle screw and cortical boundaries (arrow) for assessment of breaches. (C) 3D-rendered visualization of the pedicle screw with corresponding axial CT measurements of its length and width. (D) Axial CT scan depicting a left lateral pedicle breach, corresponding to a GRS grade of E (arrow). (E) Sagittal CT reconstruction showing the same lateral GRS grade E screw perforating (arrow). (F) Coronal CT scan confirming the lateral pedicle breach (arrow) (created with BioRender.com).

Postoperative CT scans were analyzed using high-resolution multiplanar reconstructions aligned with each screw's trajectory. Axial, sagittal, and coronal views were used to assess transverse, longitudinal, and cortical positioning. Screw dimensions were verified against operative records. Screw placement accuracy was assessed using the Gertzbein-Robbins Grading Scale (GRS), which stratifies screw positioning into five categories based on the extent of cortical breach detected on postoperative axial CT imaging (Grade A = no pedicle breach; B = < 2 mm; C = 2–4 mm; D = 4–6 mm; E = > 6 mm) (Gertzbein and Robbins, 1990) (Fig. 2 A).

Following the original classification, the primary endpoint of this study was the proportion of satisfactory surgical placements, defined as screws graded A or B (Gertzbein and Robbins, 1990). The cross-sectional CT slice showing the greatest screw deviation from the pedicle was selected for grading to ensure an accurate assessment of placement accuracy. Two independent reviewers (F.C., F.K.), blinded to the treatment group, evaluated the CT scans to determine the grade assigned to each screw. Interobserver variability was addressed by consulting a third independent reviewer (S.M.) to adjudicate discrepancies, ensuring consensus and classification reliability. At the patient level, the percentage of inaccurate screws was calculated as the ratio of inaccurate screws to the total number of screws placed per patient.

### 2.5 Outcome measures, data collection, and variables

The primary outcome was pedicle screw placement accuracy, defined by the GRS as accurate (Grades A–B) or inaccurate (Grades C–E). Secondary outcomes included revision surgery within 12 months and between-group differences in postoperative complications, hospitalization metrics, and radiation exposure.

Data were retrospectively extracted using a standardized codebook from medical records, operative reports, and imaging archives. Baseline variables included demographics, body mass index (BMI), comorbidities, American Society of Anesthesiologists (ASA) grade, Charlson Comorbidity Index (CCI) (Charlson et al., 1987), the Canadian clinical frailty scale (ranging from 1 (very fit) to 9 (terminally ill)) (Rockwood et al., 2005), primary diagnosis, and affected spinal segments. Surgical variables comprised technique, indication, treated levels, and screw count. Intraoperative parameters included anesthesia and surgical duration, blood loss, and intraoperative screw revisions.

Each pedicle screw was graded by GRS, with cortical breaches further categorized by deviation direction. Screw dimensions and cement augmentation use, including extravasation, were documented. Postoperative recovery metrics included hospital and intensive care unit (ICU) stay, time to mobilization, and early (pre-discharge) or late (≤12 months) complications. Revisions were recorded as early (≤3 months) or late (>3–12 months) and graded using the therapy-disability neurology (TDN) system (Terrapon et al., 2021). Imaging data included postoperative CT timing, indications, and confirmation of suspected diagnoses. Radiation exposure was quantified by total dose, dose-area product (DAP), and fluoroscopy duration.

### 2.6 Statistical analysis

Data was collected using Excel Version 16.01 (Microsoft, Redmond, WA, USA) and analyzed using GraphPad Prism Version 10.4 (GraphPad Software, Inc., San Diego, CA) and R (version 4.4.3; R Foundation for Statistical Computing, Vienna, Austria) using open-source libraries. Gaussian distribution was assessed via Shapiro-Wilk testing. Descriptive data are presented as means with standard deviations (SD), while outcome data are presented with the corresponding 95% confidence intervals (95% CI), based on data distribution. Group comparisons for continuous variables were conducted using an independent T-test or Mann-Whitney *U* test, depending on data distribution, and categorical variables were compared using Fisher's exact test.

To identify independent risk factors for pedicle screw inaccuracy (GRS B-E), a generalized linear mixed model (GLMM) with a binomial link function was applied (Cordemans et al., 2017). Given the hierarchical structure of the data, with multiple screws nested within individual patients, patient-specific random intercepts were included to account for within-subject correlation, thus allowing for a robust estimation of fixed effects associated with screw placement accuracy while minimizing bias from interpatient variability.

Propensity score matching (PSM) was conducted using the MatchIt package in R to mitigate baseline confounding (see Supplementary Material, Table S2). Propensity scores were estimated via logistic regression, and nearest neighbor matching was performed without replacement, applying a caliper of 0.25 standard deviations of the propensity score to minimize poor matches (Lunt, 2014). Matching was based on baseline demographics, including age, gender, BMI, active smoking, active alcohol and drug use, diabetes mellitus, osteoporosis, previous spine surgery, ASA score, CCI, and frailty index to ensure comparability between groups. Covariate balance was evaluated through standardized mean differences (SMD) and variance ratios, ensuring post-matching comparability. Further details on the PSM process, covariate balance assessments, and model diagnostics are provided in the Supplementary Material (for PSM see Figure S1-S3, and Table S3; for GLMM, see Fig. S4, and Table S4+S5). Group comparisons are primarily based on the propensity score–matched cohort. Results from the unmatched cohort are included in all tables for completeness and descriptive context. Missing data were assessed for randomness and assumed to be missing at random (MAR). A complete case analysis was performed as the low proportion of missing values did not warrant imputation. A two-sided p-value <0.05 was considered statistically significant.

## 3 Results

### 3.1 Patient characteristics

**Fig. 3:**
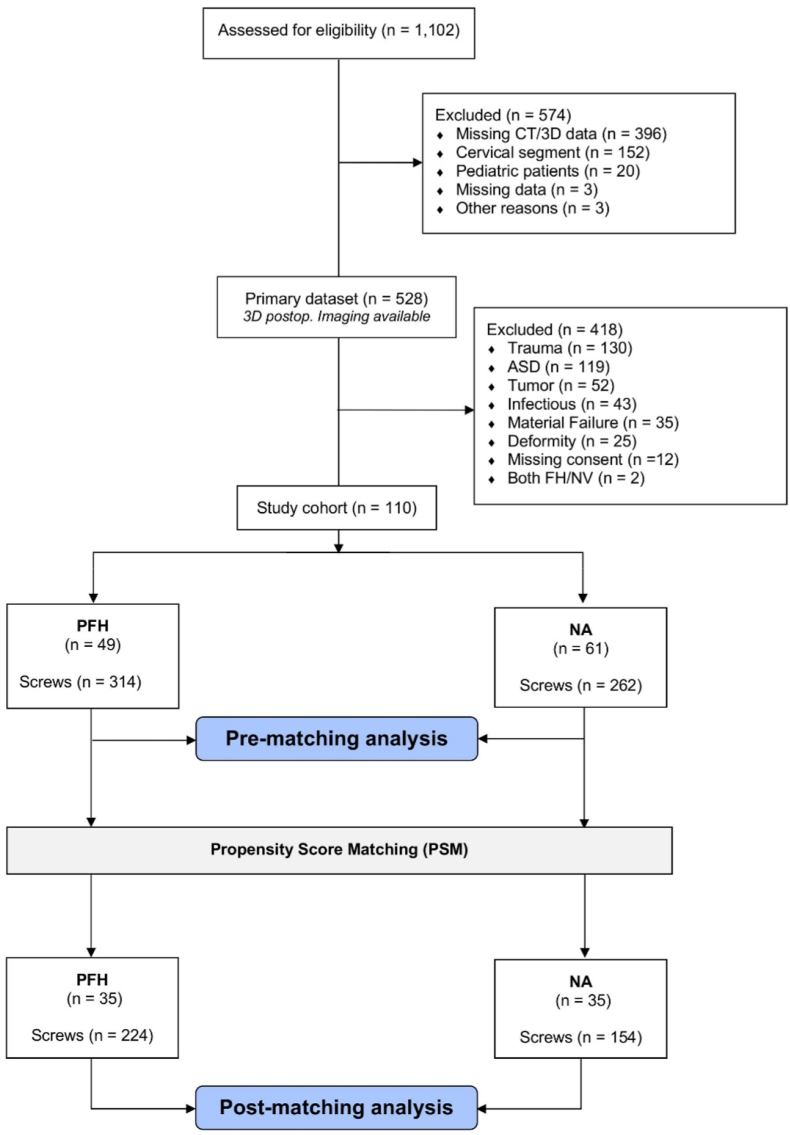
Patient selection and study cohort flowchart.

Of 1102 patients screened, 110 met the inclusion criteria (PFH, 49; NA, 61). After propensity score matching, 35 patients remained in each group (Fig. 3). After matching, groups were comparable in age (67.8 ± 11.6 vs 65.5 ± 12.8 years; p = 0.45) and comorbidities (p > 0.05). Time to mobilization was longer in PFH (1.29 ± 0.75 vs 1.00 ± 0.00 days; p = 0.04).

There was a trend toward more treated segments (3.11 ± 2.39 vs 2.03 ± 1.25; p = 0.06) and implanted screws (8.23 ± 4.77 vs 6.06 ± 2.50; p = 0.06) in the PFH group. Screw length was greater in PFH (5.06 ± 0.56 cm vs 4.88 ± 0.39 cm; p = 0.001). Cement augmentation was more frequently performed with PFH (55.8% vs 17.5%; p < 0.001), with the majority of cement-augmented screws in PFH placed in the lumbar spine (69.5%). Cement extravasation rates were low and did not differ significantly between groups similar (3.2% vs 7.4%; p = 0.29).

**Fig. 4:**
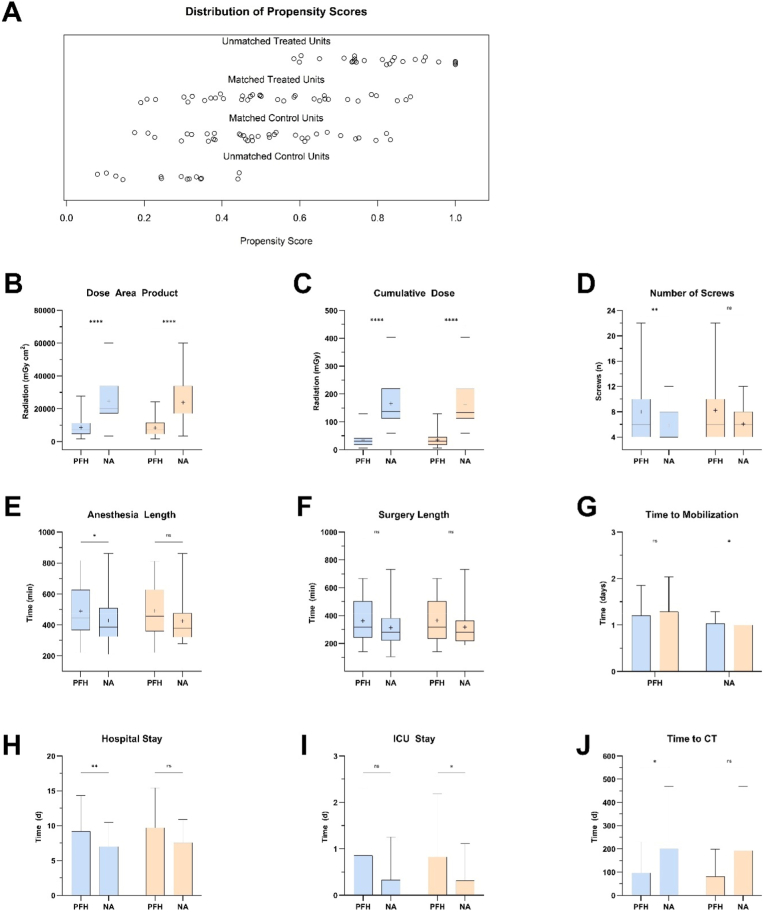
Comparison of PFH and NA pedicle screw placement before and after PSM. (A) A jitter plot demonstrating the distribution of propensity scores for PFH and NA groups before and after matching. In general, blue represents pre-matched data, while orange denotes post-matched data. (B + C) Box plots demonstrating the DAP (B) and cumulative dose (C) between PFH and NA groups which was significantly higher in NA than in PFH groups (p < 0.0001). The boxes display the distribution of values, with the horizontal line indicating the median and the plus sign (+) representing the mean. Whiskers extend to the minimum and maximum observed values. (D) Comparison between number of inserted pedicle screws. (E, F) Bar graphs comparing perioperative time parameters between PFH and NA groups. (e) Anesthesia duration was significantly longer in the PFH group (p < 0.05), while (F) surgery length showed no significant difference. Bars represent the mean with standard deviation (SD). (G–J) Bar graphs illustrating postoperative recovery and imaging timelines. (G) Time to mobilization was slightly longer in PFH (p < 0.05). (H) Hospital length of stay was significantly prolonged in PFH compared to NA (p < 0.01). (I) ICU stay was marginally longer in the NA group (p < 0.05). (J) Time to first postoperative CT scan was significantly delayed in NA (p < 0.05). Statistical significance: ns (*p* = 0.1234), *p* = 0.0332 (∗), *p* = 0.0021 (∗∗), *p* = 0.0002 (∗∗∗), *p* < 0.0001 (∗∗∗∗). *Abbreviations:* mGy, milligray; mGy·cm^2^, milligray-centimeter squared; n, number; ICU, intensive care unit; min, minutes; CT, computed tomography; PFH, power-drill fluoroscopy-controlled freehand; NA, navigation-assisted; DAP, dose-area product.

Radiation exposure was lower in PFH across total dose, dose–area product, and fluoroscopy duration (all p < 0.001), despite the higher frequency of cement augmentation. Subgroup analyses by spinal segment, hospitalization, and radiation parameters are presented in the Supplementary Material (Fig. S5–S6, Table S6–S8). Baseline characteristics are summarized in Table 1 and Fig. 4.

**Table 1:** Baseline characteristics and surgical parameters of study populations before and after matching**.**

Variable	pre-matched			post-matched		
	PFH (n = 49)	NA (n = 61)	*p*-value	PFH (n = 35)	NA (n = 35)	*p*-value
Age – year	68.80 ± 10.85	61.49 ± 13.91	**0.003**	67.77 ± 11.60	65.54 ± 12.84	0.45
Gender, female – no. (%)	28 (57.14)	29 (47.54)	0.34	19 (54.29)	19 (54.29)	>0.99
BMI – kg/m^2^	27.84 ± 3.96	28.03 ± 4.75	0.83	27.48 ± 3.75	28.47 ± 5.05	0.35
ASA Score – no. (%)	2.65 ± 0.60	2.44 ± 0.56	0.08	2.63 ± 0.60	2.49 ± 0.56	0.36
ASA 1	0 (0)	1 (1.64)	>0.99	0 (0)	0 (0)	>0.99
ASA 2	20 (40.82)	33 (54.1)	0.46	15 (42.86)	19 (54.29)	0.47
ASA 3	26 (53.06)	26 (42.62)	0.34	18 (51.43)	15 (42.86)	0.63
ASA 4	3 (6.12)	1 (1.64)	0.32	2 (5.71)	1 (2.86)	>0.99
Fraility Index – points	3.14 ± 0.84	3.28 ± 1.08	0.43	3.2 ± 0.83	3.11 ± 0.90	0.89
CCI – points	4.24 ± 2.96	3.23 ± 2.12	0.07	3.97 ± 3.10	3.71 ± 2.32	0.98
Active smoking – no. (%)	15 (30.61)	24 (39.34)	0.42	11 (31.43)	12 (34.29)	>0.99
Smoking (py)	40.38 ± 20.96	26.41 ± 16.85	0.05	37.77 ± 19.05	29.37 ± 16.78	0.31
Active alcohol use – no. (%)	9 (18.37)	12 (19.67)	>0.99	6 (17.14)	6 (17.14)	>0.99
Active drug use – no. (%)	0 (0)	4 (6.56)	0.13	0 (0)	0 (0)	>0.99
Diabetes mellitus – no. (%)	16 (32.65)	9 (14.75)	**0.04**	7 (20)	8 (22.86)	>0.99
Osteoporosis – no. (%)	5 (10.2)	5 (8.2)	0.75	4 (11.43)	3 (8.57)	>0.99
Previous spine surgery – no. (%)	15 (30.61)	24 (39.34)	0.42	11 (31.43)	10 (28.57)	>0.99
Blood loss – ml	831.63 ± 574.07	677.87 ± 700.69	0.06	817.14 ± 551.19	752.86 ± 870.42	0.19
Segments – no	3 ± 2.3	1.92 ± 1.17	**0.009**	3.11 ± 2.39	2.03 ± 1.25	0.06
Screws – no	8 ± 4.6	5.84 ± 2.35	**0.009**	8.23 ± 4.77	6.06 ± 2.5	0.06
Length of anesthesia – min	489.68 ± 161.44	428.02 ± 312.97	**0.03**	491.03 ± 167.84	425.29 ± 139.70	0.07
Length of surgery – min	362.80 ± 148.24	134.76 ± 122.80	0.07	365.54 ± 154.16	317.03 ± 130.10	0.16
Length of hospital stay – days	9.18 ± 5.14	7.00 ± 3.49	**0.004**	9.71 ± 5.71	7.57 ± 3.33	0.12
Lenth of ICU stay – days	0.86 ± 1.44	0.33 ± 0.93	**0.01**	0.83 ± 1.36	0.31 ± 0.80	0.08
Time to mobilization – days	1.20 ± 0.64	1.03 ± 0.26	0.10	1.29 ± 0.75	1.00 ± 0.00	**0.04**
Radiation dose – mGy	34.45 ± 23.26	165.55 ± 76.79	**<0.0001**	34.71 ± 24.38	162.75 ± 82.96	**<0.0001**
Radiation DAP – mGy·cm^2^	8559.00 ± 5574.13	24711.85 ± 12142.60	**<0.0001**	8378.43 ± 5302.08	23793.02 ± 13161.81	**<0.0001**
Radiation duration – seconds	72.38 ± 42.31	147.46 ± 56.66	**<0.0001**	74.63 ± 43.21	146.03 ± 55.93	**<0.0001**
Time to first CT – days	97.04 ± 132.36	200.72 ± 269.16	**0.02**	81.60 ± 117.46	192.53 ± 277.32	0.09

Data are reported as means ± standard deviations or numbers (percentages*). Abbreviations:* ASA, American Society of Anesthesiologists; BMI, body mass index; CCI, Charlson Comorbidity Index; DAP, dose-area product; ICU, intensive care unit; kg/m^2^, kilograms per square meter; mGy, milligray; mGy·cm^2^, milligray-centimeter squared; ml, milliliters; NA, navigation-assisted; no., number; PFH, power-drill fluoroscopy-controlled freehand; py, pack-years.

### 3.2 Comparative screw accuracy analysis

After matching, both techniques achieved high overall accuracy, with satisfactory screw placement (GRS Grades A–B) in 97.8% of PFH and 93.5% of NA screws (difference 4.3 percentage points, p = 0.02) (Table 2). GRS Grade A placement occurred in 92.3% and 83.1% of screws, respectively (p = 0.04; OR 0.56, 95% CI 0.30–1.04), while Grade B placement rates did not differ (7.7% vs 10.4%; p = 0.36). Severe breaches (GRS D–E) occurred only in the NA group (1.9%; p = 0.07).

**Table 2:** Screw accuracy and characteristics before and after matching.

Variable	Pre-matched			Post-matched		
	PFH (n = 314)	NA (n = 262)	*p*-value	PFH (n = 224)	NA (n = 154)	*p*-value
GRS – no. (%)						
A	281 (89.49)	214 (81.68)	**0.008**	203 (92.27)	128 (83.12)	**0.04**
B	27 (8.60)	33 (12.60)	0.13	17 (7.73)	16 (10.39)	0.36
C	6 (1.91)	8 (3.05)	0.42	4 (1.82)	5 (3.25)	0.49
D	0 (0)	3 (1.15)	0.06	0 (0)	2 (1.30)	0.17
E	0 (0)	4 (1.53)	**0.03**	0 (0)	3 (1.95)	0.07
Surgical result – no. (%)						
Satisfactory	308 (98.09)	247 (94.27)	**0.02**	220 (98.21)	144 (93.51)	**0.02**
Unsatisfactory	6 (1.91)	15 (5.73)	**0.02**	4 (1.79)	10 (6.49)	**0.02**
Screw Length – cm	5.08 ± 0.54	4.90 ± 0.44	**<0.0001**	5.06 ± 0.56	4.88 ± 0.39	**0.001**
Screw diameter – mm	6.36 ± 0.82	6.44 ± 0.78	0.176	6.32 ± 0.84	6.45 ± 0.82	0.09
Cement augmentation – no. (%)	173 (55.10)	34 (12.98)	**<0.0001**	125 (55.80)	27 (17.53)	**<0.0001**
Extravasation – no. (%)	11 (6.36)	2 (5.88)	>0.99	4 (3.209	2 (7.41)	0.29

Data are reported as means ± standard deviations or numbers (percentages*).* Statistically significant results (p < 0.05) were marked in bold font. *Abbreviations:* cm, centimeters; mm, millimeters; GRS, Gertzbein-Robbins Grading Scale; NA, navigation-assisted; no., number; PFH, power-drill fluoroscopy-controlled freehand.

### 3.3 Revision rates

Intraoperative screw revision was infrequent and similar between groups (PFH, n = 2 [5.7%]; NA, n = 3 [8.6%]; p > 0.05) (Table 3). Early revision within 3 months occurred in 4 PF H cases (11.4%) and 3 NA cases (8.6%) (p > 0.99). Revisions in the PFH group were due to pedicle fracture with cage subsidence or dislocation (n = 2), screw misplacement (n = 1), and postoperative lower extremity paresis (n = 1). In the NA group, revisions involved screw misplacement (n = 2) and retained hardware (n = 1). At 12 months, revision surgery was more frequent in the NA group (17.1% vs 5.7%; p = 0.26). At 12 months, revision surgery was more frequent in the NA group (17.1% vs 5.7%; p = 0.26). Material failure was the most frequent cause of revision (n = 8), followed by wound dehiscence and infection (n = 4) and wound infection (n = 4). Details of revision-related adverse events are provided in Supplementary Table S10.

**Table 3:** Frequencies of revision surgery in pre- and post-matched study populations.

Variable	pre-matched			post-matched		
	PFH (n = 49)	NA (n = 61)	*p*-value	PFH (n = 35)	NA (n = 35)	*p*-value
Intraop. screw revision – no. (%)	4 (8.16)	4 (6.56)	>0.99	2 (5.71)	3 (8.57)	>0.99
Early revision surgery – no. (%)	6 (12.24)	3 (4.92)	0.18	4 (11.43)	3 (8.57)	>0.99
Time to early revision surgery – d	4.33 ± 2.34	5.00 ± 4.58	0.77	4.00 ± 2.45	5.00 ± 4.58	0.72
Late revision surgery – no. (%)	11 (22.45)	16 (26.23)	0.66	6 (17.14)	10 (28.57)	0.39
Revision surgery (up to 3 months) – no. (%)	7 (14.29)	7 (11.48)	>0.99	4 (11.43)	4 (11.43)	>0.99
Revision surgery (up to 12 months) – no. (%)	4 (8.16)	9 (14.75)	0.39	2 (5.71)	6 (17.14)	0.26
Time to late revision surgery – no. (%)	97.64 ± 104.42	140.00 ± 141.89	0.68	113.50 ± 130.24	175.80 ± 164.42	0.44
TDN discharge						
TDN 3 – no. (%)	7 (14.29)	6 (9.84)	0.56	5 (14.29)	3 (5.71)	0.71

Data are reported as means ± standard deviations or numbers (percentages*).* Statistically significant results (p < 0.05) were marked in bold font. *Abbreviations:* intraop., intraoperative; NA, navigation-assisted; no., number; PFH, power-drill fluoroscopy-controlled freehand; postop., postoperative; TDN, Therapy-Disability-Neurology Grade.

## 4 Discussion

In this propensity score-matched cohort study of 70 patients undergoing pedicle screw fixation for degenerative thoracolumbar pathology, both PFH and NA techniques achieved high screw placement accuracy (>93% satisfactory placement). The absolute difference in accuracy (4.3 percentage points), while statistically significant, was modest, and both techniques exceeded accuracy rates commonly considered clinically acceptable in published literature. PFH demonstrated comparable accuracy to NA with significantly lower patient radiation exposure. These findings suggest that in selected degenerative cases with preserved anatomy, PFH may represent a viable alternative to navigation, particularly where radiation reduction is prioritized or navigation resources are limited.

### 4.1 Comparative accuracy

Both techniques achieved high screw placement accuracy, with satisfactory placement (GRS A + B) in 97.8% of PFH screws versus 93.5% of NA screws (p = 0.02). The 4.3 percentage-point difference, while statistically significant, was modest in absolute terms, with both techniques exceeding 93% accuracy. These findings align with published literature reporting PFH accuracy of 96–99% in degenerative and deformity cohorts (Faldini et al., 2024; Alomari et al., 2023), and NA accuracy of 93–100% across various platforms and anatomical regions (Gelalis et al., 2012; Papalia et al., 2024).

The NA performance in our study (93.5%), though strong, falls at the lower end of published ranges. Several technical factors likely contributed. Our institution utilizes a mobile 3D C-arm (Siemens Cios Spin) rather than intraoperative CT, which offers lower spatial resolution and may affect registration quality. Registration drift from intersegmental motion or anatomical shifts between image acquisition and screw insertion represents a known limitation of navigation systems (Glossop and Hu, 1997; Guha et al., 2019). Additionally, temporal analysis revealed greater variability in early NA cases, suggesting learning curve effects. These factors reflect implementation-specific challenges rather than inherent limitations of navigation technology. Advanced navigation platforms with real-time registration refinement or robotic assistance likely achieve higher accuracy than observed in our cohort (Motov et al., 2025).

PFH screws were modestly but significantly longer than NA screws (5.06 ± 0.56 mm vs 4.88 ± 0.39 mm, p = 0.001), likely reflecting anterior cortex palpation techniques that enable depth optimization. Longer screws engaging the anterior cortex may hypothetically enhance pullout strength (Zindrick et al., 1986; Karami et al., 2015), though screw diameter, which did not differ between groups, exerts greater influence on flexion-extension stability (Matsukawa et al., 2021). In line with this, osteoporosis was associated with reduced screw accuracy (OR 0.43, 95% CI 0.20–0.92, p = 0.03), likely reflecting compromised cortical integrity and reduced guidewire purchase in low-density bone (Rometsch et al., 2020; Zhang et al., 2021). In clinical practice, probing, drilling, and palpation of the anterior cortex must therefore be performed with particular caution in osteoporotic patients, especially in previously fractured vertebrae, where cortical morphology may be severely altered. We rely on haptic feedback when applying the freehand technique but routinely determine the intended pedicle screw length preoperatively by CT-based planning, as well, to avoid anterior breaches and associated vascular or visceral complications. Accordingly, PFH should be applied selectively in osteoporotic patients and by surgeons with sufficient experience, with careful measurement of drilling depth and heightened caution during anterior cortex probing.

Notably, cement augmentation was more frequently performed in the PFH group, predominantly at lumbar levels (69.5%), reflecting bone quality considerations rather than increased anatomical complexity. No alternative pedicle access strategies, such as funnel techniques or in-out-in trajectories, were applied in either group, as no dysplastic pedicles or anatomical variations requiring modified probing techniques were encountered.

These findings suggest that in degenerative cases with preserved anatomy, PFH achieves comparable accuracy to NA. However, navigation remains essential in complex scenarios including deformity correction, revision surgery, and distorted anatomy, where real-time guidance provides critical advantages (Mendelsohn et al., 2016).

### 4.2 Complications and revision surgery

Revision surgery rates were similar between groups at 12 months (5.7% PFH vs 17.1% NA, p = 0.26). Most revisions in both groups were prompted by complications unrelated to screw placement accuracy, including adjacent segment degeneration, hardware failure, and infection (Table S9). These complications likely reflect the natural history of degenerative disease and biomechanical factors inherent to spinal fusion (Anandjiwala et al., 2011) rather than technical placement errors, consistent with the extended interval to postoperative imaging in this cohort (Table 1). The similar revision rates despite modestly different radiographic accuracy suggest that both techniques provide clinically adequate screw placement in this patient population.

Notably, the overall adverse event and revision burden in this cohort appears higher than in some published degenerative fusion series. This may reflect a tertiary-care, real-world case mix with multilevel constructs and comorbidity, and the selective use of postoperative CT imaging (predominantly symptom-driven), which may enrich the cohort for patients with clinical concerns and inflate observed event rates relative to routine-surveillance or registry cohorts. In addition, the treatment of a frail and multimorbid population at a tertiary spine center is known to independently increase postoperative risk (Yildiz et al., 2025). Finally, with respect to the navigation cohort, residual confounding related to early adoption, learning-curve effects, and variability in system utilization cannot be excluded.

### 4.3 Radiation exposure, workflow efficiency, and economic considerations

Patient radiation exposure was 65% lower in the PFH group across all metrics (dose-area product, cumulative dose, fluoroscopy time; p < 0.0001 for all parameters), despite longer constructs and higher cement augmentation rates (Table 1). While navigation reduces occupational exposure to surgical personnel by eliminating continuous fluoroscopy, the 3D imaging acquisition required for registration contributes substantially to patient dose (Mendelsohn et al., 2016). This difference may be particularly relevant for multilevel constructs, younger patients, or settings prioritizing cumulative radiation reduction.

Operative time and fluoroscopy duration were shorter for PFH in constructs involving ≥5 segments (Figures S5-S6), suggesting workflow advantages in extensive reconstructions. Hospital length of stay did not differ significantly between groups after propensity matching, though small subgroup sample sizes limit definitive conclusions regarding procedure-specific recovery patterns.

From an institutional perspective, the two techniques differ meaningfully in capital investment. Navigation systems comparable to the platform used at our institution have reported acquisition costs of approximately USD 290,000–435,000 (Haik et al., 2025), excluding maintenance contracts and navigation-specific disposables. In contrast, mobile fluoroscopic systems suitable for power-drill freehand workflows are associated with substantially lower acquisition costs, with reported prices for Iso-C–type C-arms in the range of approximately USD 25,000–45,000 (Hüfner et al., 2007), and lower costs for older-generation models such as the system used at our institution. Economic evaluations of navigation often cite reduced reoperation rates related to pedicle screw misplacement as a potential cost offset in economic models (Dea et al., 2016). In the present cohort, however, revision surgery attributable to pedicle screw misplacement was infrequent and did not differ between groups, limiting the relevance of such assumptions. Under these circumstances, economic considerations are primarily driven by differences in capital investment and maintenance requirements rather than by revision-related savings.

### 4.4 Strengths and limitations

This study has important strengths and limitations requiring balanced interpretation. To our knowledge, it represents the first propensity-matched comparison of PFH and NA specifically in degenerative thoracolumbar pathology. Rigorous propensity score matching across 11 baseline variables achieved excellent covariate balance (standardized mean differences <0.1 for all variables post-matching), blinded dual-reviewer assessment minimized measurement bias, and hierarchical modeling appropriately accounted for within-patient correlation. Comprehensive radiation dosimetry provides clinically actionable data, and the real-world design enhances external validity.

However, the retrospective design with surgeon-dependent technique selection represents the primary limitation. The technique assignment was based on clinical judgment, anatomical considerations, and institutional workflow rather than randomization, introducing potential selection bias. Although rigorous matching balanced measured confounders, unmeasured variables, including pedicle morphology, vertebral rotation, and perceived case difficulty, cannot be excluded. Notably, pre-matching PFH cases had longer constructs and more instrumented levels, suggesting simpler cases were not systematically allocated to PFH. These differences were fully balanced after matching, and the modest absolute accuracy difference (4.3 percentage points) with high performance in both groups (>93%) suggests any residual confounding had a limited impact on comparative outcomes.

Postoperative CT imaging was obtained selectively based on institutional protocols (30% routine surveillance) or clinical indications (70% symptom-driven), creating potential ascertainment bias. However, imaging patterns were similar between groups, and among symptom-prompted scans, only 25% revealed screw-related pathology (Supplementary Table S1). Many CT scans were obtained for unrelated indications, providing an unbiased sampling. Nevertheless, asymptomatic malpositions were likely missed in both groups. Our findings, therefore, reflect comparative accuracy between techniques rather than absolute population-level accuracy, a critical distinction that does not invalidate the comparative analysis given similar imaging patterns between groups.

Learning curve effects likely influenced navigation performance, with greater variability observed in early cases. Whether this reflects temporal skill acquisition, differential surgeons’ experience, or evolving case selection cannot be determined from the retrospective data. Technique-specific surgeon experience was not quantified, representing an unmeasured confounder. Additionally, our institution utilizes a mobile 3D C-arm rather than intraoperative CT, which may have contributed to navigation accuracy (93.5%) at the lower end of published ranges. Advanced navigation platforms may achieve higher accuracy than observed in our cohort.

The single-center design limits generalisability to other institutions with different equipment, surgeon experience, and practice patterns. Our cohort comprised exclusively degenerative pathology with preserved anatomy. Therefore, findings may not extend to complex deformity or revision surgery, where navigation advantages might be more pronounced. Finally, while statistically significant, the absolute accuracy difference was modest (4.3 percentage points), and its clinical significance is uncertain given similar revision rates between groups. Thus, this study should be considered hypothesis-generating, warranting prospective validation in multicenter cohorts with standardized imaging protocols and anatomical stratification.

## 5 Conclusion

In this propensity-matched cohort of patients with degenerative thoracolumbar pathology, PFH pedicle screw placement demonstrated comparable accuracy to navigation-assisted techniques while offering significantly lower radiation exposure. These findings suggest that PFH, as a distinct drill-based insertion method, warrants further investigation as a viable alternative in selected cases, particularly where navigation is unavailable or when institutional workflow and radiation considerations are paramount. Navigation-assisted surgery remains a highly valuable technology, particularly in complex spinal procedures where enhanced visualization of challenging anatomy is essential. Prospective, multicenter trials with standardized imaging protocols are needed to validate these findings and define the optimal indications for each technique.

## Data availability statement

The datasets generated and analyzed during the current study are available upon reasonable request from the corresponding author.

## Funding

This research did not receive any specific grant from funding agencies in the public, commercial, or not-for-profit sectors.

## Conflict of interest

The authors declare no competing interests.

## Figures and Tables

**Table A1:** STROBE Statement—Checklist of items that should be included in reports of cohort studies.

	Item No	Recommendation	Page No
**Title and abstract**	1	(*a*) Indicate the study's design with a commonly used term in the title or the abstract	1 + 3
		(*b*) Provide in the abstract an informative and balanced summary of what was done and what was found	
**Introduction**			
Background/rationale	2	Explain the scientific background and rationale for the investigation being reported	7
Objectives	3	State specific objectives, including any prespecified hypotheses	7
**Methods**			
Study design	4	Present key elements of study design early in the paper	8
Setting	5	Describe the setting, locations, and relevant dates, including periods of recruitment, exposure, follow-up, and data collection	8
Participants	6	(*a*) Give the eligibility criteria, and the sources and methods of selection of participants. Describe methods of follow-up	8
		(*b*) For matched studies, give matching criteria and number of exposed and unexposed	
Variables	7	Clearly define all outcomes, exposures, predictors, potential confounders, and effect modifiers. Give diagnostic criteria, if applicable	8–9
Data sources/measurement	8∗	For each variable of interest, give sources of data and details of methods of assessment (measurement). Describe comparability of assessment methods if there is more than one group	9
Bias	9	Describe any efforts to address potential sources of bias	9
Study size	10	Explain how the study size was arrived at	9
Quantitative variables	11	Explain how quantitative variables were handled in the analyses. If applicable, describe which groupings were chosen and why	9
Statistical methods	12	(*a*) Describe all statistical methods, including those used to control for confounding	12–13
		(*b*) Describe any methods used to examine subgroups and interactions	
		(*c*) Explain how missing data were addressed	
		(*d*) If applicable, explain how loss to follow-up was addressed	
		(*e*) Describe any sensitivity analyses	
**Results**			
Participants	13∗	(a) Report numbers of individuals at each stage of study—eg numbers potentially eligible, examined for eligibility, confirmed eligible, included in the study, completing follow-up, and analyzed	14–16
		(b) Give reasons for non-participation at each stage	
		(c) Consider use of a flow diagram	
Descriptive data	14∗	(a) Give characteristics of study participants (eg demographic, clinical, social) and information on exposures and potential confounders	17–18
		(b) Indicate number of participants with missing data for each variable of interest	
		(c) Summarise follow-up time (eg, average and total amount)	
Outcome data	15∗	Report numbers of outcome events or summary measures over time	17–18
Main results	16	(a) Give unadjusted estimates and, if applicable, confounder-adjusted estimates and their precision (eg, 95% confidence interval). Make clear which confounders were adjusted for and why they were included	17–18
		(b) Report category boundaries when continuous variables were categorized	17–18
		(c) If relevant, consider translating estimates of relative risk into absolute risk for a meaningful time period	17–18
Other analyses	17	Report other analyses done—eg analyses of subgroups and interactions, and sensitivity analyses	17–18
**Discussion**			
Key results	18	Summarise key results with reference to study objectives	19–20
Limitations	19	Discuss limitations of the study, taking into account sources of potential bias or imprecision. Discuss both direction and magnitude of any potential bias	20
Interpretation	20	Give a cautious overall interpretation of results considering objectives, limitations, multiplicity of analyses, results from similar studies, and other relevant evidence	19–20
Generalisability	21	Discuss the generalisability (external validity) of the study results	19–20
**Other information**			
Funding	22	Give the source of funding and the role of the funders for the present study and, if applicable, for the original study on which the present article is based	26

Variable	Post-matched		
	PFH (n = 224)	NA (n = 154)	*p*-value
GRS – no. (%)			
A	203 (92.27)	128 (83.12)	**0.04**
B	17 (7.73)	16 (10.39)	0.36
C	4 (1.82)	5 (3.25)	0.49
D	0 (0)	2 (1.30)	0.17
E	0 (0)	3 (1.95)	0.07
Surgical result – no. (%)			
Satisfactory	220 (98.21)	144 (93.51)	**0.02**
Unsatisfactory	4 (1.79)	10 (6.49)	**0.02**
Screw Length – cm	5.06 ± 0.56	4.88 ± 0.39	**0.001**
Screw diameter – mm	6.32 ± 0.84	6.45 ± 0.82	0.09
Cement augmentation – no. (%)	125 (55.80)	27 (17.53)	**<0.0001**
Extravasation – no. (%)	4 (3.209	2 (7.41)	0.29
