# Supplementary material for: Power-drill fluoroscopy-controlled technique as an alternative to navigation-assisted pedicle screw placement: A propensity score-matched retrospective cohort study
Source: Brain Spine. 2026 Feb 2;6:105959. doi: 10.1016/j.bas.2026.105959 (PMC12905786; doi:10.1016/j.bas.2026.105959)
Supplement: Multimedia component 1 [file mmc1.docx]

**Supplementary Material**

Power-Drill Fluoroscopy-Controlled Freehand vs. Navigation-Assisted Pedicle Screw Placement: A Propensity Score-Matched Retrospective Cohort Study

Felix Corr^1^ MD, MSc.; Faizan Kareem^2^; Linda Bättig^1^, MD; Benedict Caspari^3^; Karl Kapahnke^4^; Silvio Heinig^1^, MD; Simon Behringer^1^, MD; Erik Schulz^1^, MD; Yesim Yildiz^1^, MD; Nader Hejrati^1^, MD; Oliver Bozinov^1^, MD, Prof.; Benjamin Martens^5^, MD; Kern Singh^6^, MD; Martin N. Stienen^1^, MD, Prof., FEBNS; Stefan Motov^1^, MD

**Affiliations:**

^1^ Department of Neurosurgery & Interdisciplinary Spine Center, HOCH Health Ostschweiz, Cantonal Hospital St. Gallen, Rorschacher Strasse 95, 9007 St. Gallen, Switzerland

^2^ Faculty of Medicine, University of Zürich, Rämistrasse 100, 8006 Zürich, Switzerland

^3^ Faculty of Medicine, University of Düsseldorf, Universitätsstrasse 1, 40225 Düsseldorf, Germany

^4^ Faculty of Medicine, University of Greifswald, Domstrasse 11, 17489 Greifswald, Germany

^5^ Department of Orthopedic Surgery and Traumatology & Interdisciplinary Spine Center, HOCH Health Ostschweiz, Cantonal Hospital St. Gallen, St. Gallen Switzerland

^6^ Department of Orthopaedic Surgery, Rush University Medical Center, 1611 W. Harrison St., Suite #300, Chicago, IL 60612, USA

***Corresponding Author:**

Felix Corr, MD, MSc.

Department of Neurosurgery

HOCH Health Ostschweiz

Kantonsspital St. Gallen

Rorschacherstrasse 95

9000 St. Gallen, Switzerland

Email: [felix.corr@h-och.ch](mailto:felix.corr@h-och.ch)

ORCID: 0000-0002-5365-7511

**Table of Contents**

[Table S1 3](#_Toc213403379)

[Table S2 4](#_Toc213403380)

[Figure S1. 6](#_Toc213403381)

[Table S3 7](#_Toc213403382)

[Figure S2. 8](#_Toc213403383)

[Figure S3. 9](#_Toc213403384)

[Figure S4. 10](#_Toc213403385)

[Table S4 11](#_Toc213403386)

[Table S5 12](#_Toc213403387)

[Figure S5. 13](#_Toc213403388)

[Figure S6. 14](#_Toc213403389)

[Table S6 16](#_Toc213403390)

[Table S7 17](#_Toc213403391)

[Table S8 19](#_Toc213403392)

[Table S9 20](#_Toc213403393)

[Table S10 21](#_Toc213403394)

**Table S1.** Indications for CT Scans and Confirmation of Suspicion.

| **Reason for CT** | **Proportion**  **- no. (%)** | **Confirmation of suspicion**  **– no. (%)** |
| --- | --- | --- |
| Regular FU | 33 (30.00) | 5 (15.5) |
| Other | 15 (13.64) | 2 (13.33) |
| Pain | 13 (11.82) | 3 (23.08) |
| Material failure | 12 (10.91) | 6 (50) |
| Sensomotor deficit | 10 (9.09) | 5 (50) |
| GI Disorder | 8 (7.27) | 1 (12.5) |
| Pulmonary Embolism | 7 (6.36) | 3 (42.86) |
| Staging/oncological | 4 (3.64) | 0 (0) |
| Fracture | 3 (2.73) | 2 (66.67) |
| PJK | 2 (1.82) | 1 (50) |
| Infection | 2 (1.82) | 0 (0) |
| Missing | 1 (0.91) | - |

This table presents the distribution of reasons for performing a CT scan and the proportion of cases in which the initial suspicion was confirmed. The data includes routine follow-up (FU) examinations as well as symptom-driven indications such as pain, material failure, and sensomotor deficits. Additionally, specific conditions like pulmonary embolism (PE), gastrointestinal (GI) disorders, oncological staging, fractures, and infections are reported. For regular follow-up, confirmation of suspicion refers to newly diagnosed, asymptomatic pathologies detected on imaging*. Abbreviations:* CT, computed tomography; FU, follow-up; GI, gastrointestinal; PJK, proximal junctional kyphosis.

**Table S2.** R Code for Propensity Score Matching and Generalized Linear Mixed Model (GLMM) Analysis

| **Statistical Modeling and Analysis** | **Code** |
| --- | --- |
| PSM | library(readxl)  library(dplyr)  library(MatchIt)  matching_data <- read_excel("select_file.xlsx", sheet = "Tabelle1") %>%  mutate(Group = as.factor(Group))  psa_n <- matchit(Group ~ . -ID,  data = matching_data,  distance = "glm",  method = "nearest",  m.order = "largest",  replace = FALSE,  caliper = 0.25)  print(psa_n)  summary(psa_n) |
| GLMM | library(lme4)  library(glmmTMB)  library(performance)  data <- readxl::read_excel("select_file.xlsx", sheet = 1)  data <- data %>%  mutate(  Patient_ID = as.factor(Patient_ID),  Gender = as.factor(Gender),  Active_Smoking = as.factor(Active_Smoking),  Active_Alcohol_use = as.factor(Active_Alcohol_use),  Diabetes_mellitus = as.factor(Diabetes_mellitus),  Osteoporosis = as.factor(Osteoporosis),  Previous_Surgery = as.factor(Previous_Surgery)  )  data$Accuracy <- as.numeric(as.character(data$Accuracy))  glmm_model <- glmer(  Accuracy ~ Age + Gender + BMI + Active_Smoking + Active_Alcohol_use +  Diabetes_mellitus + Osteoporosis + Previous_Surgery + (1 \| Patient_ID),  data = data,  family = binomial,  control = glmerControl(optimizer = "bobyqa")  )  summary(glmm_model)  check_collinearity(glmm_model)  check_model(glmm_model)  check_overdispersion(glmm_model)  isSingular(glmm_model)  VarCorr(glmm_model) |

PSM was conducted using the MatchIt package in R to adjust for baseline differences between PFH and NA pedicle screw placement groups. A logistic regression-based nearest-neighbor matching approach was applied, using a caliper of 0.25 to restrict poor matches. The propensity score model included all relevant covariates, excluding the patient ID. The resulting matched dataset was examined for balance using summary diagnostics. To assess determinants of pedicle screw accuracy, a GLMM was fitted using glmer from the lme4 package. The model incorporated fixed effects for demographic and clinical covariates, with Patient ID as a random intercept to account for within-subject clustering. The dependent variable, accuracy, was treated as a binary outcome (accurate vs. inaccurate). Model diagnostics were evaluated using functions from the performance package, including collinearity checks, model fit assessments, and overdispersion tests. *Abbreviations:* GLMM, generalized linear mixed model; PSM, propensity score matching.

**Figure S1.** Propensity Score Density Before and After Matching.


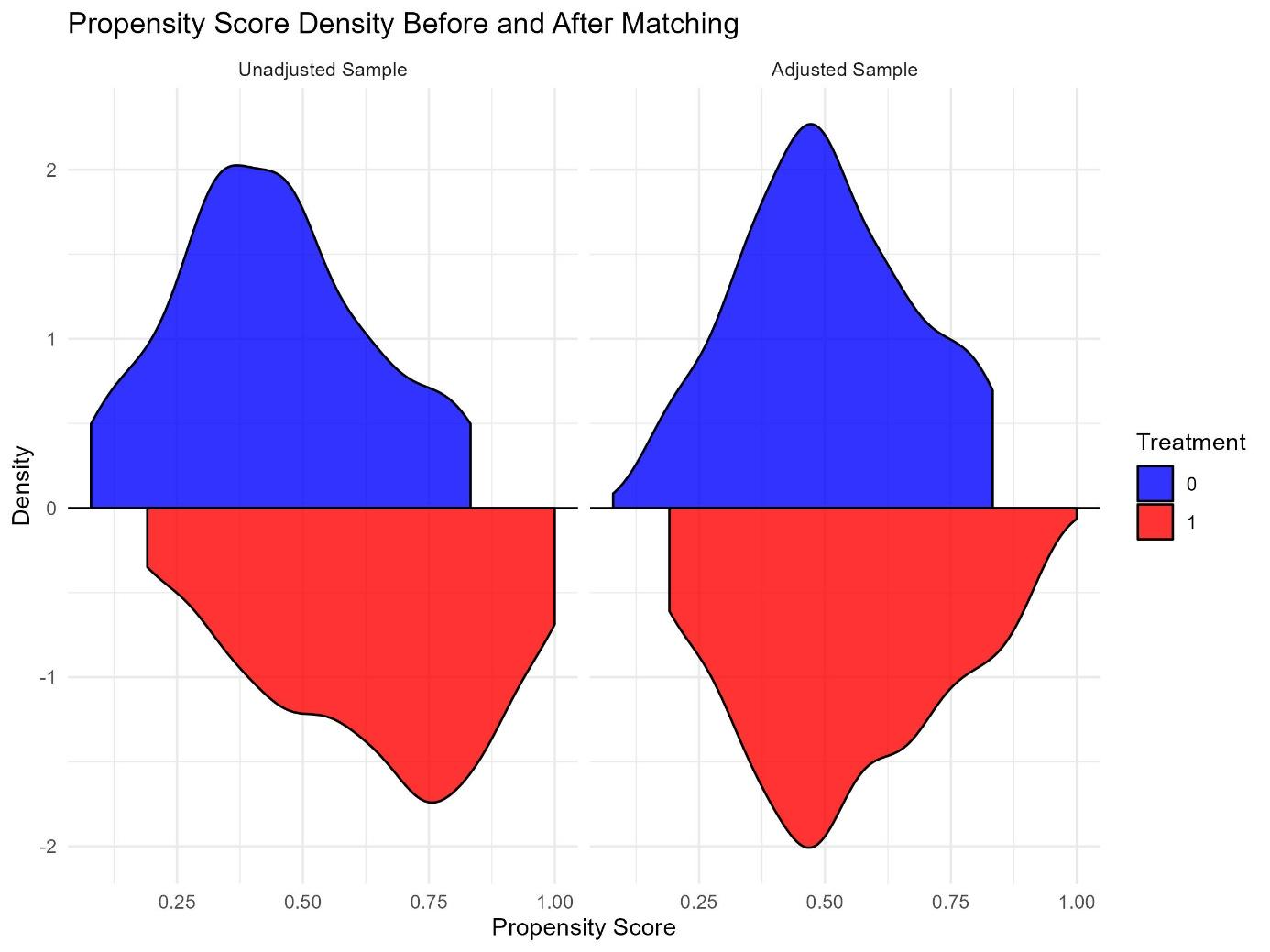


This figure illustrates kernel density estimates of propensity scores for patients undergoing PFH versus NA pedicle screw placement. The left panel (unadjusted sample) demonstrates an initial imbalance in propensity score distributions between PFH (blue) and NA (red). The right panel (adjusted sample) shows improved overlap following PSM, indicating enhanced covariate balance between groups.

**Table S3. Summary of Propensity Score Matching Characteristics.**

| Metric | Value |
| --- | --- |
| Number of Treated (PFH) | 61.000 |
| Number of Control (NA) | 49.000 |
| Number of Matched Pairs | 70.000 |
| Mean Propensity Score (Pre-Matching) | **0.5545** |
| SD Propensity Score (Pre-Matching) | 0.2309 |
| Mean Propensity Score (Post-Matching) | **0.5166** |
| SD Propensity Score (Post-Matching) | 0.1821 |
| Min Propensity Score (Pre-Matching) | 0.0795 |
| Max Propensity Score (Pre-Matching) | 10.000 |
| Min Propensity Score (Post-Matching) | 0.1752 |
| Max Propensity Score (Post-Matching) | 0.8845 |

**This table presents key metrics related to the PSM process for PFH and NA pedicle screw placement. A total of 61 PFH cases and 49 NA cases were initially available, resulting in 70 matched pairs after the matching procedure. The mean propensity score before matching was 0.5545 (SD: 0.2309), decreasing to 0.5166 (SD: 0.1821) after matching, indicating improved balance between groups. *Abbreviations:* NA, navigation-assisted; PFH, power-drill fluoroscopy-controlled freehand; SD, standard deviation.**

**Figure S2.** Covariate Balance Before and After Propensity Score Matching.


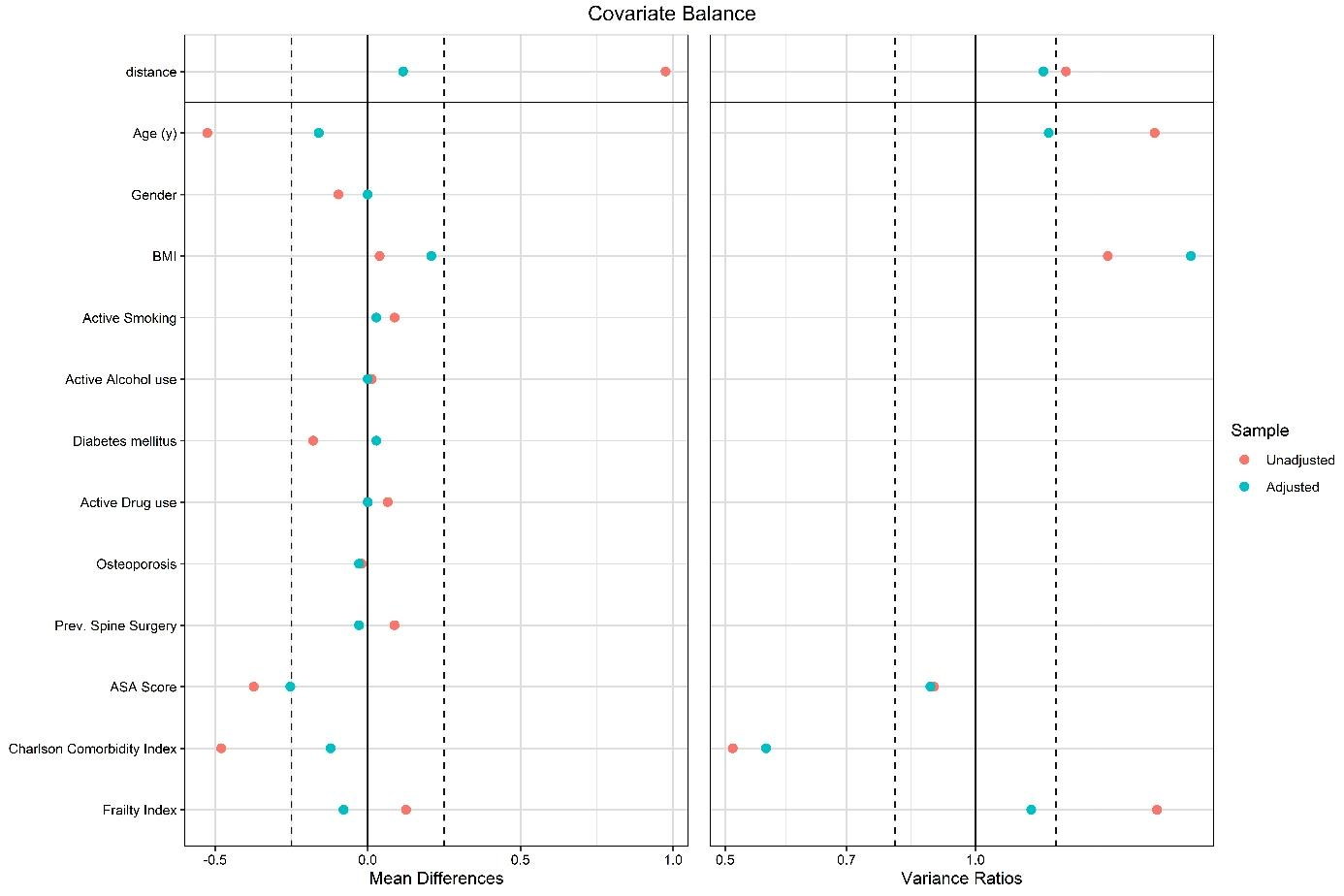


This love plot visualizes the balance of key covariates between the PFH and NA pedicle screw placement groups before (red) and after (blue) PSM. The left panel depicts standardized mean differences, where values closer to zero indicate improved balance. The right panel shows variance ratios, with values near one signifying comparable variance across groups. *Abbreviations:* ASA, American Society of Anesthesiologists Score; BMI, body mass index; Prev, previous.

**Figure S3.** Covariate Distributions in Unmatched and Matched Samples.


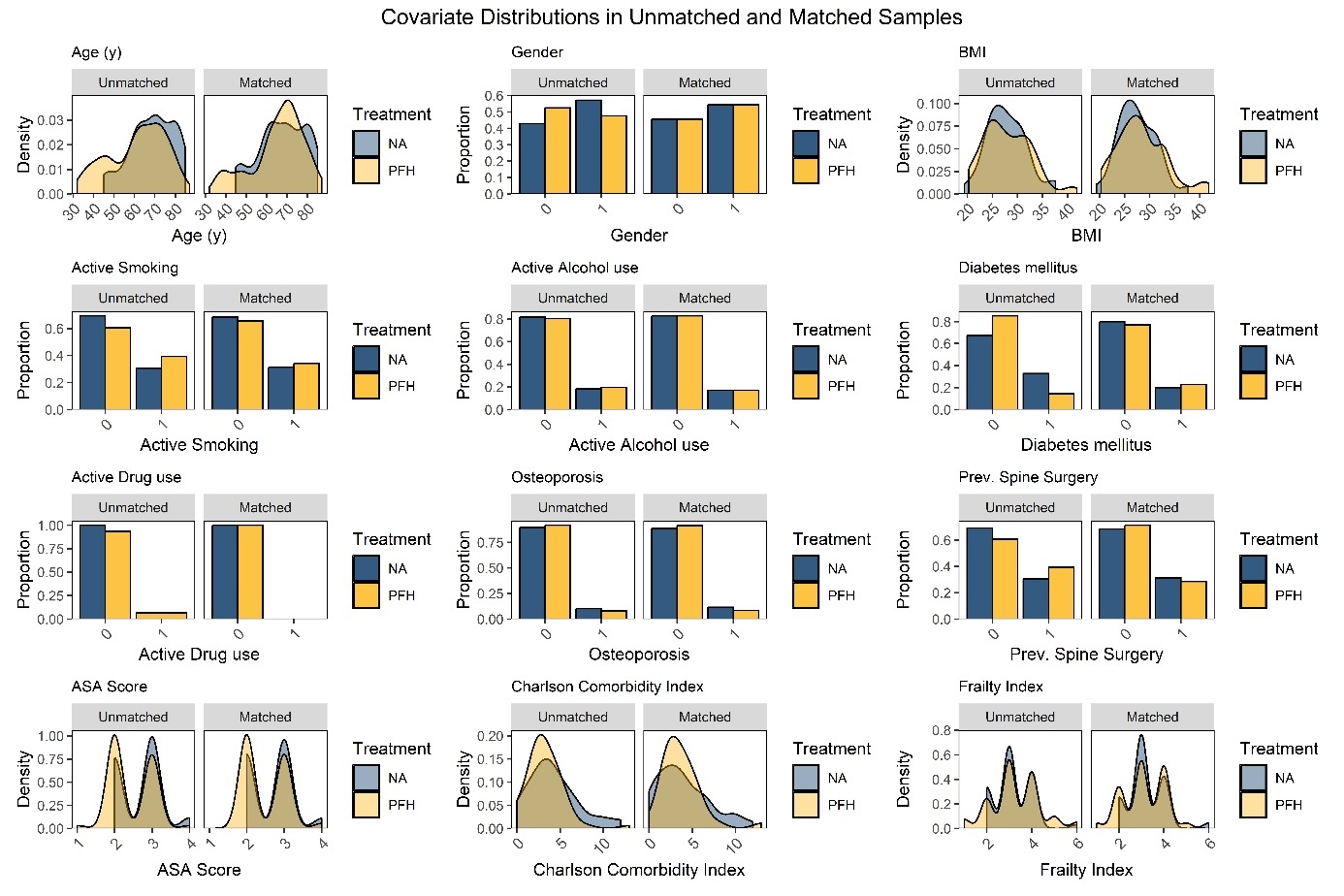


This figure compares the distribution of key covariates between the PFH (yellow) and NA (blue) pedicle screw placement groups before (unmatched) and after (matched) PSM. Density plots (for continuous variables) and bar charts (for categorical variables) illustrate covariate distributions. Prior to matching, several covariates show imbalances between treatment groups. After matching, distributions align more closely, indicating improved comparability between PFH and NA groups. *Abbreviations:* ASA, American Society of Anesthesiologists Score; BMI, body mass index; PFH, power-drill fluoroscopy-controlled freehand, NA, navigation-assisted Prev., previous; y, years.

**Figure S4. Model Diagnostics for the GLMM.**


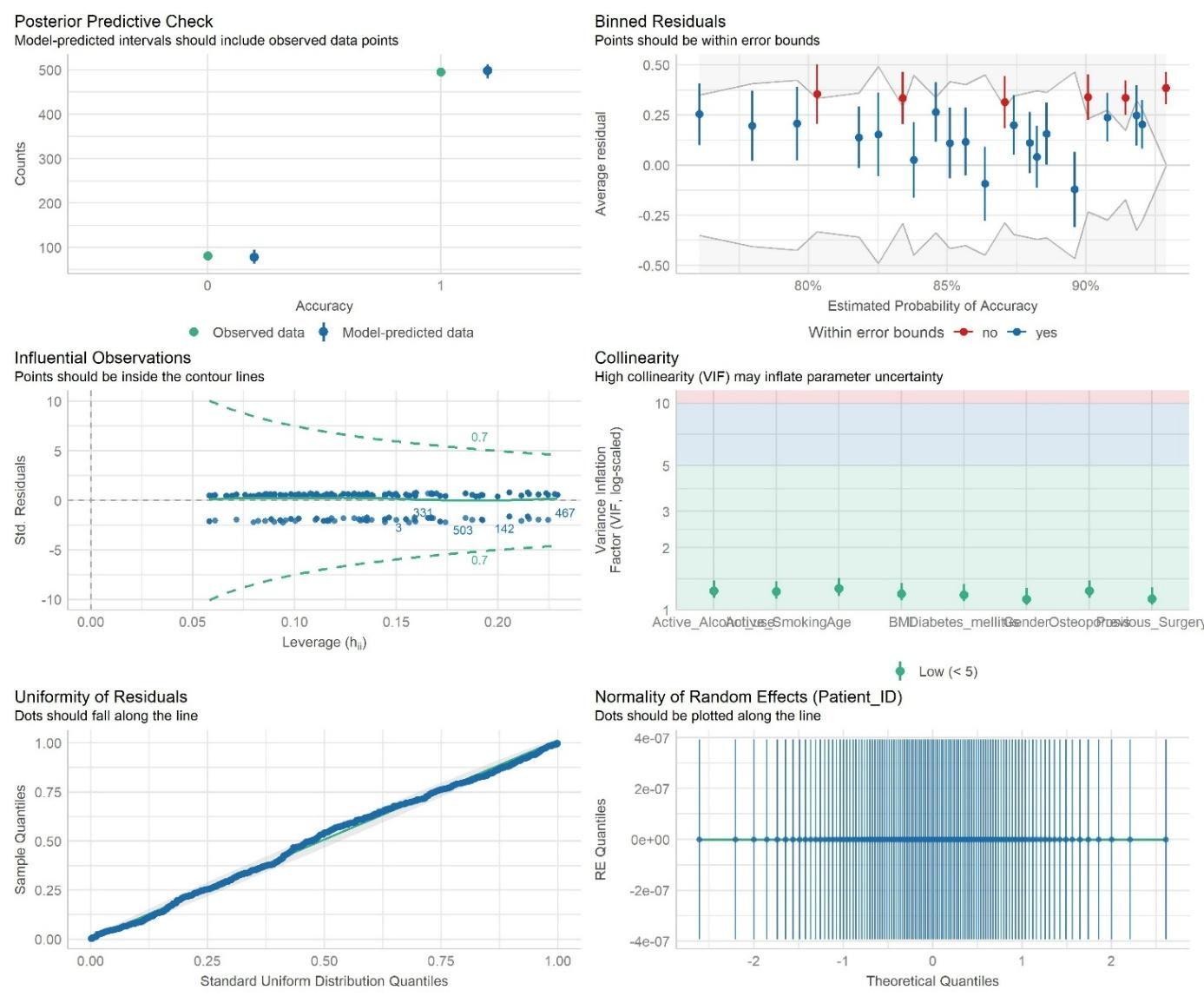


**This figure presents diagnostic plots assessing the validity of the GLMM used to estimate determinants of pedicle screw accuracy. The upper left panel (Posterior Predictive Check) compares observed (green) and model-predicted (blue) data, ensuring that predicted intervals include observed data points. The upper right panel (Binned Residuals) plots average residuals against estimated probabilities of accuracy. The middle left panel (Influential Observations) displays a leverage vs. standardized residuals plot to identify influential data points. The middle right panel (Collinearity, VIF) assesses multicollinearity among predictors, with Variance Inflation Factor (VIF) values below 5 indicating low collinearity and reduced parameter uncertainty. The lower left panel (Uniformity of Residuals) presents a quantile-quantile (Q-Q) plot comparing residuals to a standard uniform distribution. The lower right panel (Normality of Random Effects) examines the normality of random effects (Patient_ID) through a Q-Q plot, with points ideally following the reference line.**

**Table S4.** Variance Inflation Factor (VIF) Analysis for Multicollinearity Assessment in the GLMM.

| Term | VIF | VIF 95% CI | Increased SE | Tolerance | Tolerance 95% CI |
| --- | --- | --- | --- | --- | --- |
| Age | 1.69 | [1.53, 1.91] | 1.3 | 0.59 | [0.52, 0.65] |
| Gender | 1.2 | [1.12, 1.35] | 1.1 | 0.83 | [0.74, 0.90] |
| BMI | 1.23 | [1.14, 1.39] | 1.11 | 0.81 | [0.72, 0.88] |
| Smoking | 1.33 | [1.22, 1.49] | 1.15 | 0.75 | [0.67, 0.82] |
| Alcohol | 1.37 | [1.26, 1.54] | 1.17 | 0.73 | [0.65, 0.80] |
| Diabetes mellitus | 1.56 | [1.42, 1.76] | 1.25 | 0.64 | [0.57, 0.71] |
| Osteoporosis | 1.28 | [1.18, 1.44] | 1.13 | 0.78 | [0.69, 0.85] |
| Prev. Surgery | 1.24 | [1.15, 1.40] | 1.12 | 0.8 | [0.72, 0.87] |
| ASA Score | 2.02 | [1.81, 2.30] | 1.42 | 0.49 | [0.44, 0.55] |
| Charlson Comorbidity Index | 2.45 | [2.17, 2.79] | 1.56 | 0.41 | [0.36, 0.46] |
| Frailty Index | 1.46 | [1.33, 1.65] | 1.21 | 0.68 | [0.61, 0.75] |

This table presents the Variance Inflation Factor (VIF) values and associated statistics for covariates included in the GLMM assessing determinants of pedicle screw accuracy. A VIF below 5 indicates low multicollinearity, reducing the risk of inflated standard errors and biased parameter estimates. The ASA Score (VIF = 2.02) and CCI (VIF = 2.45) exhibit the highest VIF values but remain within acceptable limits. Tolerance values, the inverse of VIF, further support sufficient independence of covariates, with all values above 0.40, indicating low collinearity. *Abbreviations:* ASA Score, American Society of Anesthesiologists Score; BMI, body mass index; SE, standard error; VIF, variance inflation factor; 95% CI, 95% confidence interval.

**Table S5.** Correlation Matrix of Fixed Effects in the GLMM.

|  | Age | Gender | BMI | Smoking | Alcohol | DM | Osteoporosis | Prev. Surgery | ASA | CCI |
| --- | --- | --- | --- | --- | --- | --- | --- | --- | --- | --- |
| Age |  |  |  |  |  |  |  |  |  |  |
| Gender | -0.052 |  |  |  |  |  |  |  |  |  |
| BMI | 0.041 | -0.053 |  |  |  |  |  |  |  |  |
| Smoking | 0.229 | 0.011 | 0.216 |  |  |  |  |  |  |  |
| Alcohol | -0.210 | 0.319 | 0.019 | -0.059 |  |  |  |  |  |  |
| DM | 0.253 | 0.057 | -0.289 | -0.096 | -0.144 |  |  |  |  |  |
| Osteoporosis | -0.201 | -0.026 | 0.130 | 0.065 | 0.236 | 0.074 |  |  |  |  |
| Prev. Surgery | -0.042 | 0.158 | -0.116 | -0.059 | 0.091 | -0.088 | 0.157 |  |  |  |
| ASA | 0.028 | -0.174 | -0.078 | -0.186 | -0.176 | -0.020 | -0.041 | -0.184 |  |  |
| CCI | -0.426 | 0.126 | 0.147 | 0.211 | 0.214 | -0.385 | 0.096 | 0.276 | -0.481 |  |
| FI | -0.072 | -0.098 | 0.014 | -0.100 | -0.165 | -0.094 | -0.161 | -0.065 | -0.334 | -0.013 |

This table presents the correlation structure among fixed effects in the GLMM assessing determinants of pedicle screw accuracy. Moderate correlations exist between some covariates, notably age and BMI (r = -0.655) and CCI and ASA Score (r = -0.481), reflecting expected clinical relationships. Other notable correlations include age and CCI (r = -0.426) and active smoking and CCI (r = 0.211). Despite these associations, the correlations remain within acceptable limits, suggesting that collinearity is unlikely to significantly distort model estimates. *Abbreviations:* ASA, American Society of Anesthesiologists score; BMI, body mass index; CCI, Charlson comorbidity index; DM, diabetes mellitus; FI, frailty index; Prev., previous.

**Figure S5. Dose Area Product Before and After Propensity Score Matching stratified by Segments.**


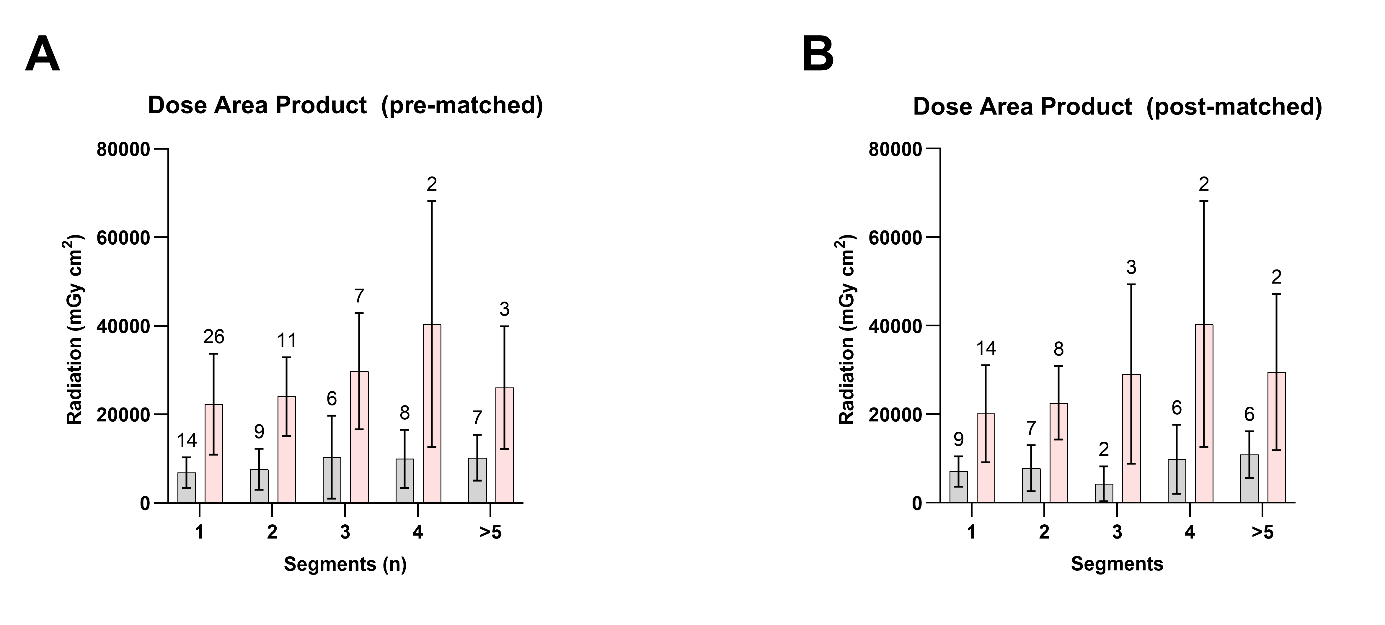


This figure compares radiation exposure, measured as Dose Area Product (DAP) in mGy·cm², across different numbers of treated spinal segments for PFH and NA pedicle screw placement. (A) DAP values before PSM, showing consistently higher radiation exposure in the NA group (pink bars) across all segment categories compared to PFH (gray bars). (B) displays post-matching results, where the trend remains unchanged, with NA demonstrating higher radiation exposure, particularly in multi-segment procedures. The number of cases per segment category (e.g., sample size) is indicated above each bar. *Abbreviations:* DAP, dose area product; mGy, milligray; cm², square centimeter; n, number.

**Figure S6. Associations Between Number of Segments and Perioperative Parameters Before and After PSM.**


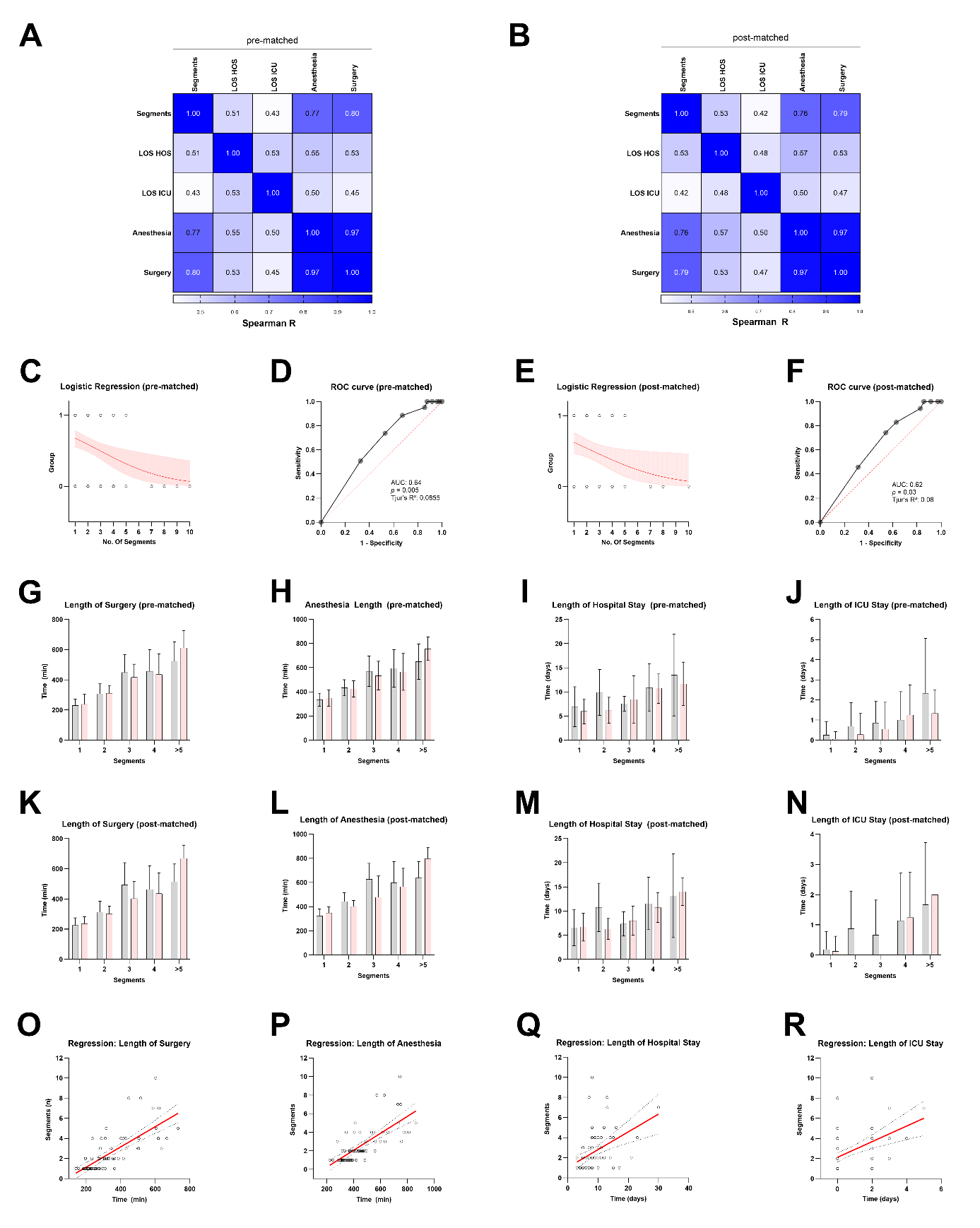


**This figure explores the relationship between the number of treated spinal segments and various perioperative parameters for PFH and NA pedicle screw placement. Panels A and B present Spearman correlation heatmaps before (pre-matched) and after (post-matched) PSM, respectively, illustrating correlations between the number of segments and perioperative variables such as length of surgery, anesthesia duration, and length of hospital and ICU stays. Panels C and E display logistic regression models assessing the predictive relationship between the number of segments and treatment group assignment, while Panels D and F present the corresponding receiver operating characteristic (ROC) curves, indicating the classification performance before and after matching. Panels G–J and K–N compare the length of surgery, anesthesia duration, hospital stay, and ICU stay across different segment categories before and after matching, respectively. Panels O–R depict linear regression analyses (post-matched) examining the relationship between the number of treated segments and perioperative variables. *Abbreviations:* AUC, area under the curve; HOS, hospital stay; ICU, intensive care unit; LOS, length of stay; min, minutes; ROC, receiver operating characteristic.**

**Table S6.** Logistic Regression Model Parameters for Predicting Treatment Group Assignment Based on the Number of Segments

| Parameter | pre-matched | post-matched |
| --- | --- | --- |
| Î²0 | 1.104 | 0.83 |
| Î²1 | -0.3752 | -0.3341 |
| X at 50% | 2.944 | 2.484 |
| Std. Error (Î²0) | 0.3615 | 0.4391 |
| Std. Error (Î²1) | 0.133 | 0.1539 |
| Std. Error (X at 50%) | 0.5876 | 0.7479 |
| 95% CI Î²0 | 0.4239 to 1.849 | -0.0003754 to 1.735 |
| 95% CI Î²1 | -0.6598 to -0.1350 | -0.6699 to -0.05900 |
| 95% CI X at 50% | 1.876 to 4.898 | -0.003484 to 5.124 |
| Odds Ratio Î²0 | 3.018 | 2.293 |
| Odds Ratio Î²1 | 0.6872 | 0.716 |
| 95% CI Odds Ratio Î²0 | 1.528 to 6.354 | 0.9996 to 5.671 |
| 95% CI Odds Ratio Î²1 | 0.5170 to 0.8737 | 0.5118 to 0.9427 |
| \|Z\| | 2.821 | 2.171 |
| P value (Slope) | 0.0048 | 0.0299 |
| Log-likelihood Ratio (GÂ²) | 10.12 | 5.852 |
| P value (LRT) | 0.0015 | 0.0156 |
| Area under ROC Curve | 0.637 | 0.6241 |
| Std. Error (AUC) | 0.0538 | 0.06698 |
| 95% CI (AUC) | 0.5316 to 0.7425 | 0.4928 to 0.7554 |
| P value (AUC) | 0.0138 | 0.0742 |
| Tjur's RÂ² | 0.08552 | 0.07573 |
| Cox-Snell's RÂ² | 0.08791 | 0.0802 |
| Model Deviance (GÂ²) | 141.1 | 91.19 |
| Equation | log odds = 1.104 - 0.3752*X | log odds = 0.8300 - 0.3341*X |

This table summarizes key parameters from the logistic regression models assessing the predictive relationship between the number of treated spinal segments and treatment group assignment (PFH vs. NA pedicle screw placement) before (pre-matched) and after (post-matched) PSM. *Abbreviations:* AUC, area under the curve; CI, confidence interval; G², log-likelihood ratio statistic; LRT, likelihood ratio test; OR, odds ratio; ROC, receiver operating characteristic; SE, standard error; Std. Error, standard error; Tjur's R², coefficient of discrimination; |Z|, absolute value of the Z-score.

**Table S7.** Stratified Comparison of Perioperative Parameters by Number of Segments.

| **Length surgery pre-matched** | | | | | | |
| --- | --- | --- | --- | --- | --- | --- |
| Segments | P value | Mean rank of PFH | Mean rank of NA | Mean rank diff. | Mann-Whitney U | q value |
| 1 | 0,863342 | 24,50 | 23,74 | 0,7581 | 240,0 | 0,975296 |
| 2 | 0,965640 | 12,60 | 12,43 | 0,1714 | 69,00 | 0,975296 |
| 3 | 0,757692 | 9,000 | 8,111 | 0,8889 | 28,00 | 0,975296 |
| 4 | 0,825175 | 7,222 | 6,500 | 0,7222 | 16,00 | 0,975296 |
| >5 | 0,547619 | 4,500 | 6,000 | -1,500 | 6,000 | 0,975296 |
| **Surgery post-matched** | | | | | | |
| Segments | P value | Mean rank of PFH | Mean rank of NA | Mean rank diff. | Mann-Whitney U | q value |
| 1 | 0,990319 | 14,05 | 13,97 | 0,07670 | 87,50 | >0,999999 |
| 2 | 0,587047 | 10,31 | 8,850 | 1,463 | 33,50 | 0,988196 |
| 3 | 0,400000 | 4,333 | 2,667 | 1,667 | 2,000 | 0,988196 |
| 4 | 0,787879 | 6,286 | 5,500 | 0,7857 | 12,00 | 0,994697 |
| >5 | 0,142857 | 3,667 | 7,000 | -3,333 | 1,000 | 0,721429 |
| **Anesthesia pre-matched** | | | | | | |
| Segments | P value | Mean rank of PFH | Mean rank of NA | Mean rank diff. | Mann-Whitney U | q value |
| 1 | 0,966191 | 22,86 | 23,06 | -0,2074 | 215,0 | >0,999999 |
| 2 | 0,763766 | 13,05 | 12,11 | 0,9429 | 64,50 | >0,999999 |
| 3 | >0,999999 | 8,571 | 8,444 | 0,1270 | 31,00 | >0,999999 |
| 4 | 0,825175 | 7,222 | 6,500 | 0,7222 | 16,00 | >0,999999 |
| >5 | 0,380952 | 4,333 | 6,333 | -2,000 | 5,000 | >0,999999 |
| **Anesthesia post-matched** | | | | | | |
| Segments | P value | Mean rank of PFH | Mean rank of NA | Mean rank diff. | Mann-Whitney U | q value |
| 1 | 0,650394 | 12,60 | 14,06 | -1,463 | 71,00 | 0,795758 |
| 2 | 0,211070 | 11,31 | 8,050 | 3,263 | 25,50 | 0,673333 |
| 3 | 0,400000 | 4,333 | 2,667 | 1,667 | 2,000 | 0,673333 |
| 4 | 0,787879 | 6,286 | 5,500 | 0,7857 | 12,00 | 0,795758 |
| >5 | 0,285714 | 3,833 | 6,500 | -2,667 | 2,000 | 0,673333 |
| **LOS HOS pre-matched** | | | | | | |
| Segments | P value | Mean rank of PFH | Mean rank of NA | Mean rank diff. | Mann-Whitney U | q value |
| 1 | 0,590710 | 25,50 | 23,23 | 2,274 | 224,0 | 0,997976 |
| 2 | **0,015219** | 16,55 | 9,607 | 6,943 | 29,50 | 0,076858 |
| 3 | 0,975524 | 8,571 | 8,444 | 0,1270 | 31,00 | 0,997976 |
| 4 | 0,690909 | 6,667 | 7,750 | -1,083 | 15,00 | 0,997976 |
| >5 | 0,988095 | 5,083 | 4,833 | 0,2500 | 8,500 | 0,997976 |
| **LOS HOS post-matched** | | | | | | |
| Segments | P value | Mean rank of PFH | Mean rank of NA | Mean rank diff. | Mann-Whitney U | q value |
| 1 | 0,581233 | 12,95 | 14,72 | -1,764 | 76,50 | 0,942667 |
| 2 | **0,018328** | 12,75 | 6,900 | 5,850 | 14,00 | 0,092557 |
| 3 | 0,800000 | 3,167 | 3,833 | -0,6667 | 3,500 | 0,942667 |
| 4 | 0,933333 | 5,857 | 6,250 | -0,3929 | 13,00 | 0,942667 |
| >5 | 0,571429 | 4,167 | 5,500 | -1,333 | 4,000 | 0,942667 |
| **LOS ICU pre-matched** | | | | | | |
| Segments | P value | Mean rank of PFH | Mean rank of NA | Mean rank diff. | Mann-Whitney U | q value |
| 1 | 0,541166 | 25,44 | 23,26 | 2,179 | 225,0 | 0,861678 |
| 2 | 0,272069 | 13,95 | 11,46 | 2,486 | 55,50 | 0,861678 |
| 3 | 0,509615 | 9,429 | 7,778 | 1,651 | 25,00 | 0,861678 |
| 4 | 0,853147 | 6,778 | 7,500 | -0,7222 | 16,00 | 0,861678 |
| >5 | 0,738095 | 5,250 | 4,500 | 0,7500 | 7,500 | 0,861678 |
| **LOS ICU post-matched** | | | | | | |
| Segments | P value | Mean rank of PFH | Mean rank of NA | Mean rank diff. | Mann-Whitney U | q value |
| 1 | >0,999999 | 14,23 | 13,84 | 0,3835 | 85,50 | >0,999999 |
| 2 | 0,068627 | 11,38 | 8,000 | 3,375 | 25,00 | 0,346569 |
| 3 | >0,999999 | 4,000 | 3,000 | 1,000 | 3,000 | >0,999999 |
| 4 | >0,999999 | 5,857 | 6,250 | -0,3929 | 13,00 | >0,999999 |
| >5 | 0,892857 | 4,333 | 5,000 | -0,6667 | 5,000 | >0,999999 |

Mann-Whitney U tests were performed to compare surgery duration, anesthesia duration, length of hospital stay (LOS HOS), and length of ICU stay (LOS ICU) between PFH and NA pedicle screw placement groups, stratified by the number of treated spinal segments. The rank-based Mann-Whitney U test was applied using two-stage step-up correction (Benjamini, Krieger, and Yekutieli) for multiple comparisons. For each segment category, p-values were calculated to assess statistical significance, alongside mean rank values for PFH and NA, the difference in mean ranks, and the Mann-Whitney U statistic. To account for multiple comparisons, q-values were computed using FDR correction. A trend toward a longer hospital stay in the two-segment group (p = 0.015 pre-matching, p = 0.018 post-matching) was observed, but this did not retain statistical significance after multiple testing correction. No other perioperative parameter showed meaningful differences between treatment groups across segment categories. *Abbreviations:* HOS, hospital stay; ICU, intensive care unit; LOS, length of stay; NA, navigation-assisted; PFH, power-drill fluoroscopy-controlled freehand.

**Table S8.** Simple Linear Regression Models Stratified by Number of Segments.

| Parameter | Length of Surgery | Length of Anesthesia | LOS HOS | LOS ICU |
| --- | --- | --- | --- | --- |
| Slope | 0.01006 | 0.009122 | 0.1764 | 0.7756 |
| Y-intercept | -0.8633 | -1.581 | 1.047 | 2.128 |
| X-intercept | 85.78 | 173.3 | -5.933 | -2.744 |
| 1/Slope | 99.36 | 109.6 | 5.668 | 1.289 |
| Std. Error (Slope) | 0.001126 | 0.001061 | 0.04529 | 0.1877 |
| Std. Error (Y-intercept) | 0.4165 | 0.513 | 0.4462 | 0.2374 |
| 95% CI Slope | 0.007817 to 0.01231 | 0.007004 to 0.01124 | 0.08605 to 0.2668 | 0.4011 to 1.150 |
| 95% CI Y-intercept | -1.695 to -0.03220 | -2.605 to -0.5568 | 0.1563 to 1.937 | 1.654 to 2.602 |
| 95% CI X-intercept | 4.028 to 140.7 | 78.01 to 236.1 | -21.63 to -0.6099 | -5.930 to -1.574 |
| R squared | 0.5402 | 0.5244 | 0.1825 | 0.2008 |
| Sy.x | 1.344 | 1.371 | 1.792 | 1.772 |
| F-statistic | 79.89 | 73.87 | 15.18 | 17. Aug |
| P value | <0.0001 | <0.0001 | 0.0002 | 0.0001 |
| Equation | Y = 0.01006*X - 0.8633 | Y = 0.009122*X - 1.581 | Y = 0.1764*X + 1.047 | Y = 0.7756*X + 2.128 |

This table summarizes the key parameters of simple linear regression models assessing the relationship between the number of treated spinal segments and surgery duration, anesthesia duration, length of hospital stay (LOS HOS), and length of ICU stay (LOS ICU). All models demonstrated statistically significant slopes (p < 0.001), confirming a positive association between the number of treated segments and each outcome. The length of surgery and anesthesia models exhibited the highest R² values (0.5402 and 0.5244, respectively), suggesting strong linear relationships, whereas LOS HOS and LOS ICU showed weaker associations (R² = 0.1825 and 0.2008, respectively), indicating greater variability in these measures. *Abbreviations:* CI, confidence interval; HOS, hospital stay; ICU, intensive care unit; LOS, length of stay; SE, standard error; Sy.x, standard error of the estimate.

**Table S9.** GLMM analysis of baseline factors associated with pedicle screw accuracy.

| Variabl | OR | 95% CI | *p*-value |
| --- | --- | --- | --- |
| **Osteoporosis** | 0.43 | 0.20 - 0.92 | **0.03** |
| Smoking | 0.71 | 0.42 - 1.20 | 0.20 |
| Alcohol use | 0.73 | 0.40 - 1.34 | 0.31 |
| BMI | 0.97 | 0.92 - 1.03 | 0.31 |
| Age | 1.02 | 0.99 - 1.04 | 0.13 |
| Gender | 1.03 | 0.63 - 1.71 | 0.89 |
| Diabetes mellitus | 1.12 | 0.60 - 2.08 | 0.73 |
| Previous surgery | 1.21 | 0.72 - 2.04 | 0.48 |

Statistically significant results (p < 0.05) were marked in bold font. *Abbreviations:* BMI, body mass index; CI, confidence interval; GLMM, generalized linear mixed model; OR, odds ratio.

**Table S10.** Early and Late Postoperative Revision Surgeries.

| ID | Group | Early postop. complication | Time to early revision surgery (days) | Late postop. complication | Time to late revision surgery  (days) |
| --- | --- | --- | --- | --- | --- |
| 41 | PFH | screw misplacement, hematoma evacuation | 1 |  |  |
| 14 | PFH | Lower extremity paresis | 3 |  |  |
| 23 | PFH | pedicle fracture, PJK | 4 |  |  |
| 27 | PFH | cage disclocation | 4 |  |  |
| 13 | PFH | material failure | 7 |  |  |
| 45 | PFH | pedicle fracture | 7 |  |  |
| 4 | PFH |  |  | wound revision | 13 |
| 1 | PFH |  |  | wound dehiscence and infection | 16 |
| 42 | PFH |  |  | wound dehiscence and infection | 24 |
| 40 | PFH |  |  | wound revision | 40 |
| 44 | PFH |  |  | adjacent segment fracture | 42 |
| 24 | PFH |  |  | material failure | 62 |
| 18 | PFH |  |  | material failure | 77 |
| 47 | PFH |  |  | ASD | 120 |
| 8 | PFH |  |  | material failure | 135 |
| 12 | PFH |  |  | material failure with segmental instability | 176 |
| 3 | PFH |  |  | PJK | 369 |
| 100 | NA | retained hardware component | 1 |  |  |
| 75 | NA | screw misplacement | 4 |  |  |
| 54 | NA | screw misplacement, wound dehiscense, wound infection | 10 |  |  |
| 65 | NA |  |  | spondylodiscitis | 10 |
| 74 | NA |  |  | wound infection, hematoserum | 11 |
| 73 | NA |  |  | spinal abscess | 15 |
| 97 | NA |  |  | material failure | 19 |
| 64 | NA |  |  | pseudarthrosis, wound infection, sepsis | 21 |
| 67 | NA |  |  | foraminal stenosis | 31 |
| 76 | NA |  |  | material failure | 34 |
| 85 | NA |  |  | spinal abscess | 130 |
| 59 | NA |  |  | PJK and fracture | 134 |
| 82 | NA |  |  | material failure, pseudarthrosis | 136 |
| 55 | NA |  |  | material failure | 160 |
| 99 | NA |  |  | material failure | 196 |
| 95 | NA |  |  | material failure | 224 |
| 110 | NA |  |  | ASD | 241 |
| 66 | NA |  |  | material failure | 415 |
| 58 | NA |  |  | wound dehiscence and infection | 463 |

This table presents an overview of early and late postoperative complications following surgery, along with the time to revision surgery (in days) where applicable. Early complications include screw misplacement, hematoma evacuation, lower extremity paresis, pedicle fractures, and material failures. Late complications include wound infections, pseudarthrosis, ASD, PJK, and material failures. The time to revision surgery is recorded to provide insight into the progression and management of complications. Among the recorded early and late postoperative complications, material failure was the most frequent (n = 8), followed by wound dehiscence and infection (n = 4) and wound infection (n = 4). Screw misplacement (n = 3), pedicle fracture (n = 3), PJK (n = 3), and wound revision (n = 3) were also commonly observed. Less frequent complications included spinal abscess (n = 2), pseudarthrosis (n = 2), ASD (n = 2), hematoma evacuation (n = 1), lower extremity paresis (n = 1), cage dislocation (n = 1), adjacent segment fracture (n = 1), retained hardware component (n = 1), wound dehiscense (n = 1), spondylodiscitis (n = 1), hematoserum (n = 1), sepsis (n = 1), foraminal stenosis (n = 1), fracture (n = 1), and material failure with segmental instability (n = 1). *Abbreviations:* ASD, adjacent segment disease; NA, not assigned; PJK, proximal junctional kyphosis; PFH, post-fracture hospitalization.
